# Tailoring Na^+^ and Cl^−^-Selective Colorimetric Optode Arrays for Wearable Sweat Analysis: Composition Optimization and Measurement Conditions

**DOI:** 10.3390/s26185774

**Published:** 2026-09-11

**Authors:** Vasiliy S. Syutkin, Ivan P. Gryazev, Daria A. Chetverikova, Andrey V. Kalinichev, Maria A. Peshkova

**Affiliations:** 1 Institute of Chemistry, Saint Petersburg State University, 7/9 Universitetskaya Emb., St. Petersburg 199034, Russia; st087670@student.spbu.ru (V.S.S.); iv.gryazev@gmail.com (I.P.G.); d4verikova@gmail.com (D.A.C.); 2 Department of Biology—Microbiology, Aarhus University, Ny Munkegade 114, 8000 Aarhus, Denmark; akalinichev@aias.au.dk

**Keywords:** polymeric optodes, ion-selective optical sensors, chromoionophore, optode arrays, colorimetry, digital color analysis, signal robustness, artificial sweat, sweat test, cystic fibrosis

## Abstract

**Highlights:**

- Screening of 15 chromoionophore-based compositions identified optimal Na^+^- and Cl^−^-selective optodes for wearable sweat analysis in a range of 5–100 mmol/L with hysteresis < 0.1 log units, and recoveries > 93% in artificial sweat.
- Smartphone imaging with digital color analysis proved superior robustness compared to laboratory configurations, while TiO_2_-doped opaque films effectively eliminated colored-sample interference without compromising sensitivity or kinetics.
- The sensor platform establishes an analytical basis for point-of-care diagnosis of cystic fibrosis, bypassing the need for bulky clinical instrumentation, and its compatibility with high-end smartphones enables screening outside specialized laboratories.
- Beyond medical diagnostics, the tunable ion-selective system suits diverse on-site applications, e.g., environmental, food, and industrial analytics where portability outweighs spectroscopic precision.

**Abstract:**

Sweat testing is central to cystic fibrosis diagnosis, but conventional analysis depends on clinical instrumentation, creating a need for portable point-of-care alternatives. As Part 1 of this two-part study, we systematically optimized Na^+^- and Cl^−^-selective colorimetric optodes and their measurement protocols for potential integration into a wearable device for in situ sweat analysis. Fifteen chromoionophore-based sensor compositions were screened over the physiologically relevant range of 5–100 mmol/L. Candidate optodes were selected based on stability in NaCl solutions and artificial sweat, hysteresis below 0.1 log units, and equilibration times under 15 min. Their analytical performance was evaluated by spectrophotometry and digital color analysis using smartphones and research-grade cameras, with a robustness parameter used to quantify signal reliability under different imaging conditions. A simple smartphone setup provided more robust performance than the tested laboratory imaging configurations. Incorporating light-scattering TiO_2_ particles into the PVC matrix produced opaque films that significantly reduced interference from colored samples without compromising sensitivity or response kinetics. Validation in artificial sweat yielded recoveries above 93% across pH 5.5–8.0. These results establish optimized sensor compositions and measurement conditions for colorimetric Na^+^ and Cl^−^ determination in sweat and provide the analytical basis for wearable-device development in Part 2.

## 1. Introduction

Ionophore-based optical sensors have provided a solution to reversible ion sensing, exploiting a variety of formats and materials. Non-invasive wearable devices based on optodes are utilized as skin patches for analyzing sweat composition [1,2,3,4,5] or as devices for detecting key ions in various biological fluids through contact with minimal sample volumes [6,7,8,9]. Optode-based devices may become a viable alternative to traditional methods of diagnosing and monitoring various diseases, of which cystic fibrosis (CF) is one of the most severe.

CF is a hereditary disorder caused by mutations in the CFTR gene, which impair the function of exocrine glands. This impairment leads to inflammatory processes in various organs, with the respiratory system and pancreas being most severely affected [10]. The severity of CF is closely related to the age of onset of symptoms, so early diagnosis is important to prevent complications and ensure optimal development. The sweat test remains the primary diagnostic method, based on the stimulation of sweat glands via pilocarpine iontophoresis and the subsequent measurement of sodium and chloride ion concentrations, also known as the Gibson and Cooke method [11]. A positive diagnosis is indicated when ion concentrations exceed 60–70 mmol/L.

To date, the standard sweat test presents notable limitations, particularly in neonates, due to insufficient sweat production caused by immature thermoregulatory mechanisms. In addition, the procedure is time consuming and requires specialized laboratory conditions, often necessitating repeat testing after the first month of life, which delays definitive diagnosis. Furthermore, sweat tests must be performed regularly to determine the effectiveness of cystic fibrosis therapy. Consequently, the development and refinement of rapid, reliable, and accessible sweat analysis techniques are considered essential for improving early detection and management of CF. Colorimetric optical sensors as part of wearable sensing patches can become a viable alternative to the existing diagnostic instruments.

Although optical signals have conventionally been recorded spectrophotometrically, digital colorimetry with portable detecting devices, such as digital cameras [12,13,14,15], single-board computers [16,17], tablet scanners [18], smartphones [19,20,21] or smartphone-based assemblies [22,23], has proven to be a successful replacement for bulky equipment.

Digital color analysis (DCA) is routinely utilized to process the digital colorimetry readouts. DCA offers high throughput, short analysis time, and on-site applicability. Furthermore, the digitized color data is easily interpreted using built-in software or automated algorithms for optical signal correction and conversion [16,18,24]. The accuracy of DCA is determined by the adequate choice of the reading device, lighting conditions and color space for data analysis.

RGB color space is predominantly used in digital color analysis of optical sensors; however, even this widely used model has a variety of subtypes that can exhibit discrepancies close to 20% [25]. The CIE Lab color space is gaining popularity, as shown in reports [15,26,27]. Several studies have been devoted to a comprehensive comparison of various color models for use in the field of chemical sensing. RGB, HSB, CIE Lab, CIEXYZ, and YCbCr color spaces were evaluated in ref. [12] for the quantitative assessment of ochratoxin A in beverages. The authors of [26] proposed a randomized approach to selecting three optimal color channels among the RGB, CIELab, and HSV color coordinates. In refs. [28,29], the relationship between the intensity values of spectrophotometric bands and color channels was established, taking into account gamma correction and RGB spectral sensitivity.

Regarding the lighting conditions suitable for acquiring a colorimetric signal, it is highly desirable for the emission spectrum of the illumination source to overlap significantly with the absorption spectra of the corresponding detectable molecule. Therefore, the use of sources with a continuous emission spectrum (incandescent lamps, xenon lamps or high-quality white LEDs) is optimal. However, caution should be exercised when using simpler devices, such as a smartphone flash; as shown in [30], typical white smartphone LEDs with a yellow luminophore may not include red bands with wavelengths above 640 nm and have a gap in the blue region (ca. 490 nm). Meanwhile, a number of papers on luminescent optodes [12,13,31] report the use of narrow-band LEDs with emissions corresponding to absorption bands of the luminophore. In refs. [32,33], this approach was implemented in tandem with a grayscale camera to acquire the signal of chromoionophore-based polymeric optodes. Several illumination systems were critically evaluated in ref. [34] for processing optode colors in the HSV color space.

However, there is no unified approach to optimizing the measurement and processing setup that includes a proper light source, a suitable reading device, and the most informative color coordinates for an arbitrary colorimetric system thus far. Moreover, in real environments, DCA readings can be biased by the sample matrix, inherent sample color, biofouling, and unstable lighting conditions. All these factors hinder the reproducibility and standardization of measurements. There have been a few reports aimed at simultaneously addressing some of these issues. In ref. [33], two measurement assemblies were compared: a white LED with a color camera and an array of narrow-band red, green and blue LEDs in combination with a grayscale CCD matrix, with the latter setup proving to be more effective. Furthermore, the authors of [35] proposed reducing the optode transparency and eliminating color interference from whole blood samples by introducing inert scattering TiO_2_ microparticles into the sensor matrix. Ref. [32] conducts a comparative study of two combinations using incandescent lamps with a digital camera and red, green and blue LEDs with a monochrome camera, as well as a critical evaluation of normalized RGB and HSV color spaces for use in DCA.

In this work, we present a large-scale screening of the available optode compositions suitable for measuring sodium and chloride ions simultaneously in a physiological range typical of human sweat fluid and potentially applicable as a sensing component of wearable devices for sweat analysis in patients with cystic fibrosis. We evaluate and compare the impact of various lighting fixtures, recording devices, and other determining factors on the optical signal under conditions of in situ measurement, using the robustness parameter proposed in [32]. The theoretical and experimental exchange/coextraction constants of the studied optodes are compared, and the range of operation, response sensitivity, time, and hysteresis of the obtained response curves are thoroughly evaluated. The resulting optode arrays are validated in simulated sweat fluid.

## 2. Materials and Methods

### 2.1. Reagents and Solutions

9-(Diethylamino)-5-[(2-octyldecyl)imino]benzo[a]phenoxazine (ETH 5350, Ch III), 9-dimethylamino-5-[4-(16-butyl-2,14-dioxo-3,15-dioxaeicosyl)phenylimino]benzo[a]phenoxazine (ETH 2439, Ch II), 3-octadecanoylimino-7-(diethylamino)-1,2-benzophenoxazine (ETH5294, Ch I), sodium tetrakis[3,5-bis(1,1,1,3,3,3-hexafluoro-2-methoxy-2-propyl)phenyl]borate trihydrate (NaHFPB), potassium tetrakis(4-chlorophenyl)borate (KT*p*ClPB), 4-*tert*-butylcalix[4]arene-tetraacetic acid tetraethyl ester (sodium ionophore X, Na X), bis[(12-crown-4)methyl] dodecylmethylmalonate (sodium ionophore VI, Na VI), 4,5-dimethyl-3,6-dioctyloxy-o-phenylene-bis(mercurytrifluoroacetate) (chloride ionophore II, Cl II, ETH9009), bis(2-ethylhexyl) sebacate (DOS), tetrahydrofuran (THF), cyclohexanone (CH), high molecular weight poly(vinyl chloride) (PVC) were Selectophore Grade reagents from Sigma-Aldrich (Burlington, MA, USA). Titanium(IV) oxide nanopowder (21 nm primary particle size, TEM) and analytical grade 4-(2-hydroxyethyl)piperazine-1-ethanesulfonic acid (HEPES) were also from Sigma-Aldrich (Burlington, MA, USA). Inorganic reagents HNO_3_, KOH, NaCl, NaH_2_PO_4_·H_2_O were analytical grade, obtained from Reaktiv, St. Petersburg, Russia. Organic reagents urea, lactic acid (80%), nitrilotriacetic acid, and tris(hydroxymethyl)aminomethane (TRIS buffer solution) were analytical grade or puriss. p.a. reagents from NevaReaktiv (St. Petersburg, Russia).

The following materials were used as substrates for the sensor arrays: cover glass for spin coating (1 mm thick, Fisher Scientific, Pittsburgh, PA, USA), Teflon transparent film F-4MB or Teflon non-transparent sheet (Formoplast, St. Petersburg, Russia) for drop-cast arrays, and polypropylene (PP) adhesive PCR plate seals (50 μm thick, Servicebio, Wuhan, China).

Freshly deionized water (resistance of 18.2 MOhm cm, GWB1, Beijing Purkinje General Instrument Co., Beijing, China) was used throughout this work. NaCl calibration solutions with a fixed pH value and varied NaCl concentrations were obtained from two stock solutions (1 and 3 mol/L) by sequential dilution using the 0.01 mol/L HEPES solution. Artificial sweat solutions were prepared from the standard background components listed in Table S1 [36,37] and a weighed portion of NaCl to achieve a concentration of 1 mol/L. This stock solution was then diluted with the blank artificial sweat containing only the background components. Thus, a series of 9–10 solutions with concentration increments of 10 mmol/L was prepared (Table S2). The pH values were adjusted with small aliquots of 1 mol/L HNO_3_ or KOH solutions.

### 2.2. Apparatus

A spin coater (Spin150, POLOS, Colorado Springs, CO, USA) and a 3D printer (IronBio X1, St. Petersburg, Russia) were used to prepare the optode films. The optical response of the thin films was recorded using a spectrophotometer (Shimadzu UV-1800, Kyoto, Japan). The signal of the optode arrays was registered with various image capturing devices: a digital research-quality camera MS-5 mounted on an optical microscope LOMO MSP-2 with software MCview v. 4.11.18042.20201128 (all obtained from LOMO Microsystems, St. Petersburg, Russia); a professional digital camera Canon EOS 1100D, with a camera lens Canon EF-S 18–55 mm *f*/4–5.6 IS STM (all obtained from Canon Inc., Tokyo, Japan); a Xiaomi Redmi Note 8 Pro, 2019 (Xiaomi Corp., Beijing, China; **smartphone 1**); an iPhone SE (1st gen), 2019 (Apple Inc., Cupertino, CA, USA; **smartphone 2**); and a Huawei P60 Pro, 2023 (Huawei Technologies Co. Ltd., Shenzhen, China; **smartphone 3**).

The TiO_2_ nanoparticles were dispersed in a solvent using either a BioSan Combi-Spin FVL-2400N vortex centrifuge (2800 rpm, BioSan, Riga, Latvia) or an ultrasonic bath Stegler 10DT (Stegler, Moscow, Russia).

The pH of the solutions was controlled with a 781 pH/Ion Meter with a combined pH electrode and an integrated Pt1000 temperature sensor (both Metrohm, Herisau, Switzerland).

### 2.3. Optode Fabrication

To fabricate Na^+^ and Cl^−^-selective optode compositions, weighed amounts of PVC and DOS were dissolved in THF. An 8-fold volumetric excess of THF relative to the combined mass of matrix (unless otherwise specified) was used. Subsequently, aliquots of the solutions of the respective active components (chromoionophore, C; ionophore, L; and ion-exchanger, R) in cyclohexanone were introduced until the required component concentrations were achieved. All 15 studied optode compositions are listed in Table S3.

Compositions containing the light-scattering agent were prepared by adding different quantities of TiO_2_ nanoparticle powder to the appropriate volume of THF. The resulting suspension was then dispersed either on the vortex mixer or in the sonication bath. Following TiO_2_ dispersion, the matrix and active membrane components were introduced into the THF suspension. Prior to drop casting, the resulting mixture in a vial was shaken again until visual homogenization was achieved. For these studies, a transparent and flexible polypropylene substrate was used to form drop-cast arrays.

For the spectrophotometric study of the optode response, optode films were prepared using the spin-coating technique (7500 rpm), for which 15 µL of the liquid optode composition was dispensed onto a spinning substrate, resulting in the formation of a 1.5–3 µm thick film. To study the optode response using macrophotography, an array of optodes (9–16 sensors) was deposited on a substrate using a 3D printer. After evaporation of the solvent from 0.1 µL droplets of optode solution in THF, the films with a thickness of 15–20 µm were obtained. An example of the resulting array is shown in Figure S1A.

### 2.4. Signal Acquisition and Processing

To investigate the response of the thin optode films, the substrate coated with an optode film was placed in a 10 mm quartz cell (Yixing Purshee Optical Elements Co., Yixing, China) filled with a calibration solution, and the absorption spectrum was recorded in the range 400–750 nm. Then, the cell was refilled with the next solution without changing the position of the optode film. Typically, calibrations were performed in order of increasing NaCl concentration. Conventional baseline correction algorithms were applied to the obtained spectra. Examples of the series of spectra for the optodes with all three used chromoionophores are shown in Figure S1B–D. The photometric accuracy was ±0.002 AU, and the photometric repeatability was 0.001 AU.

The intensities of the absorption bands corresponding to the protonated forms of the chromoionophores were used as the analytical signal. Equation (1) was used to convert the absorbance at the band maximum, A, into the relative signal α, the fraction of the deprotonated chromoionophore:

(1) $$\alpha =\frac{A-A_{P}}{A_{D}-A_{P}}$$

where A_P_ and A_D_ are the absorbances of the optode film corresponding to the fully protonated and fully deprotonated states of the chromoionophore, respectively. These values were obtained by equilibrating the film with 1 mol/L HNO_3_ and 1 mol/L KOH, respectively.

The 3D-printed optode arrays were equilibrated with a droplet of the calibration solution for the time required to reach equilibrium, and then photographed using a microscope camera or other devices under fixed illumination from incandescent lamps (Natural light, 60 W, Philips, Piła, Poland), unless otherwise specified. To verify the establishment of equilibrium, additional experiments were conducted to assess the response time of the drop-cast optode membranes. The dependence of the colorimetric signal on time upon changing the solution was approximated by the decaying exponential, and the time to reach 95% of the color transition (t_95%_) was calculated from the characteristic decay time and used thereafter as equilibration time.

The obtained photographs were processed using ImageJ v. 1.53c. The image area containing an individual sensor spot was selected as a region of interest (ROI). The values of Red (R), Green (G), and Blue (B) color components, averaged over all pixels within the ROI, were obtained. To mitigate the influence of lighting fluctuations, white balance correction was applied by dividing the raw RGB values by the respective values of a white reference area around the sensors, according to Equation (2):

(2) $$R_{w}=255\frac{R}{R_{\mathrm{wb}}},\;G_{w}=255\frac{G}{G_{\mathrm{wb}}},\;B_{w}=255\frac{B}{B_{\mathrm{wb}}}$$

where R_w_, G_w_, and B_w_ are the average intensities of the color components after the color correction, and R_wb_, G_wb_, and B_wb_ are the average intensities of the white standard.

The ratios of color components, R_w_/G_w_ or R_w_/B_w_, were typically employed as the analytical signal for constructing the calibration curves, since these ratios provided the maximum response span.

In some cases, the sensor response was normalized to 1, where 1 corresponds to the optical signal of a fully deprotonated optode, and 0 is the signal of a fully protonated optode. The values of 0 and 1 were assigned to the colorimetric signal of the optodes equilibrated with 1 mol/L HNO_3_ and 1 mol/L KOH, respectively.

To plot calibration curves, the experimental points were approximated either by linear regression (for a narrow concentration range) or by the sigmoid Boltzmann function (for a broader concentration range), as in Equation (3):

(3) $$y=A_{2}+\frac{A_{1}-A_{2}}{1+e^{\frac{x-x_{0}}{\mathrm{dx}}}}$$

where A_2_ and A_1_ are the upper and lower horizontal asymptotes, x_0_ is the abscissa of the inflection point of the sigmoid, dx is the time constant related to the slope of the tangent at the inflection point of the response curve. The fitting parameters and their standard errors were calculated using orthogonal distance regression.

To quantify the reliability of different colorimetric protocols, the robustness parameter, Rob, proposed by Tiuftiakov et al. [32], was employed in this study. It is defined within a normalized spherical RGB color space as the ratio of the dynamic range (in radians) to the geometric mean of the standard deviations (in radians) at each calibration point, determined across the entire concentration range (Equation (4)):

(4) $$\mathrm{Rob}=\frac{\mathrm{dyn\_range}(\mathrm{rad})}{\mathrm{st\_dev}(\mathrm{rad})}$$

All experimental data were processed using OriginPro 2023 software (OriginLab Corporation, Northampton, MA, USA).

## 3. Results and Discussion

### 3.1. Composition Optimization

The choice of the optode active components (chromoionophores and ionophores) was guided by available literature data. ETH5294 (Ch I), ETH2439 (Ch II) and ETH5350 (Ch III) were used as chromoionophores, as they have been best studied for physiological concentration ranges. The widely studied and well-characterized ionophores Na VI and Na X, with known stability constants for complexes with the sodium cation in plasticized PVC, were used to fabricate sodium-selective sensors. Among the four commercially available chloride-selective ionophores (Cl I–Cl IV), two (Cl II and Cl III) are structurally similar organomercury compounds. The Cl I ionophore is a colored metalloporphyrin, which hampers its use in optical sensors due to its intense absorption in the visible range. The Cl II ionophore appears to be the best studied among existing chloride ionophores and was chosen for this work. It is well known that, along with the nature of the optode components, its response depends on the quantitative composition [38], therefore, it was also varied in this study to finely tune the optical signal for the required concentration range.

**Figure 1:**
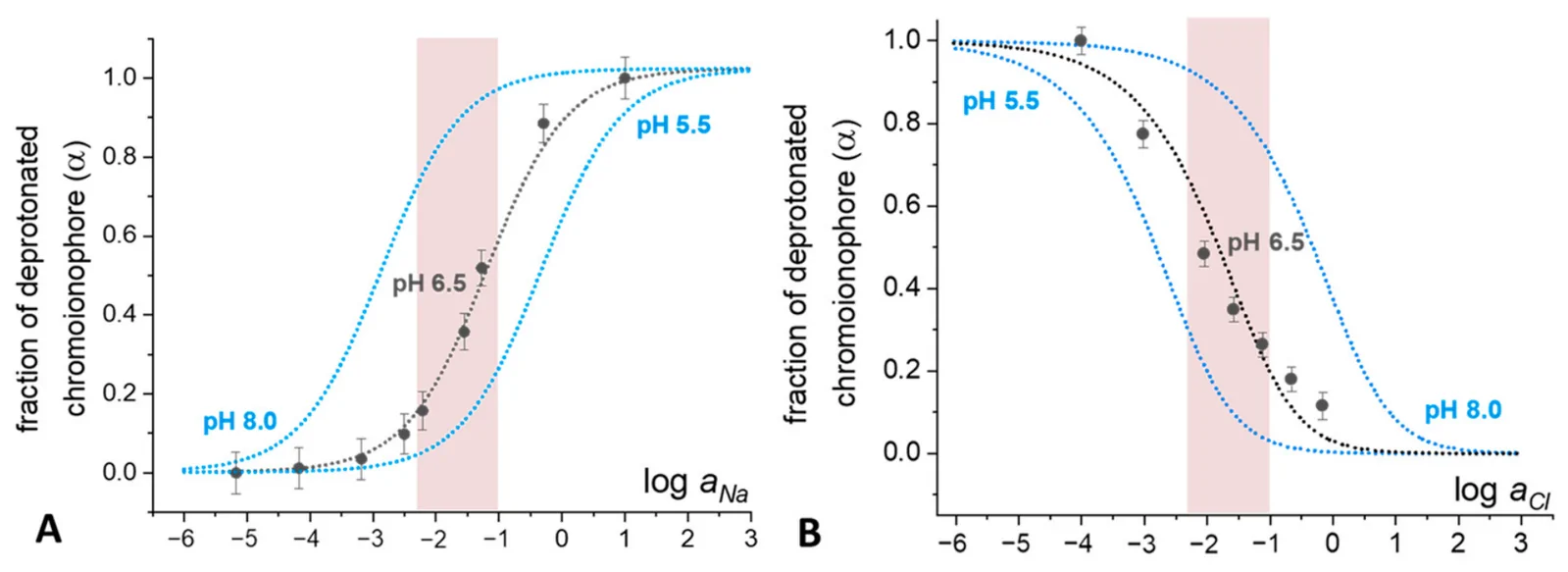
The spectrophotometric responses of the optodes of compositions 2 (**A**) and 11 (**B**) to sodium and chloride activities in the solution, respectively. pH = 6.5, symbols—experimental data, black lines—calculations with Equations (5) (**A**) and (6) (**B**) using the listed parameters and logβXL(Cl II) = 4.17. Blue lines—extrapolations of the curves computed at pH 6.5 to pH 5.5 and 8.0. Colored rectangle—the concentration range of interest. The error bars represent indirect measurement error estimated from photometric repeatability.

Optode thin films of compositions **1–14** (Table S3) were fabricated by spin coating. Their responses in NaCl solutions of different concentrations at fixed pH = 6.5 were recorded spectrophotometrically. Examples of the series of spectra for the optodes with all three used chromoionophores are shown in Figure S1B–D. The most representative examples of the corresponding response curves are presented in Figure 1 (for compositions **2** and **11**) and in Figure S2A–H. The analytical characteristics of the optical response are given in Table S4.

The theoretical response curves for the optodes of the studied compositions were calculated using the conventional formalism of the optode response in Equation (5) (for sodium-selective ion-exchange-based optodes) or Equation (6) (for chloride-selective coextraction-based optodes) [39]:

(5) $$\frac{a_{I}}{a_{H}}={\left(K_{\mathrm{exch}}\right)}^{-1}\left(\frac{\alpha }{1-\alpha }\right)\frac{R_{T}^{\text{-}}-\left(1-\alpha \right)C_{T}}{\left[L_{T}-\left(R_{T}^{\text{-}}-\left(1-\alpha \right)C_{T}\right)\right]},\;\mathrm{with}\;K_{\mathrm{exch}}=\frac{k_{I}}{k_{H}}K_{a}\beta_{\mathrm{IL}},$$

(6) $$a_{X}{\;a}_{H}={\left(K_{\mathrm{coex}}\right)}^{-1}\left(\frac{1-\alpha }{\alpha }\right)\left(\frac{\left(1-\alpha \right)C_{T}}{L_{T}-\left(1-\alpha \right)C_{T}}\right),\;\mathrm{with}\;K_{\mathrm{coex}}=k_{X}{\;k}_{H}\frac{\beta_{\mathrm{XL}}}{K_{a}},$$

where a_H_, a_I_, and a_X_ denote the activities of the respective ions in the aqueous phase; K_exch_ and K_coex_ stand for optode ion exchange and coextraction constant, respectively; α = [C]⁄C_T_ is the relative optode signal (the fraction of the deprotonated chromoionophore); C_T_, L_T_, and R^-^_T_ are the gross concentrations of the active components chromoionophore, ionophore and ion-exchanger (when needed), respectively; K_a_ is the protonation constant of the chromoionophore in plasticized PVC; β_IL(XL)_ is the ion–ionophore complex formation constant; and k_H_, k_I_, and k_X_ are Eisenman partition coefficients of the corresponding ions [40] between polymeric and aqueous phases.

The following parameters available from the literature were used for calculations: constants of ion–ionophore complexation in the polymeric phase (PVC:DOS 1:2) logβ_IL_(Na VI) = 6.55 [41], logβ_IL_(Na X) = 7.5 [42], logβ_XL_(Cl II) = 3.6 [43]; acidity constants for the chromoionophores pKa(Ch I) = 12.0, pK_a_(Ch II) = 10.2, pK_a_(Ch III) = 13.4 [44]. The cation/anion partition coefficients between the aqueous and polymeric phases, log(k_Na_/k_H_) = −0.4 (estimated for optode membranes with Ch I, [45]), −0.02 (estimated for optode membranes with Ch II, [42]) or −0.11 (estimated for optode membranes with Ch III, [42]), log(k_Cl_k_H_) = −9.37 [46]. The calculations were performed at pH 6.5 characteristic of neutral sweat fluid typically secreted by apocrine glands and then extrapolated by means of Equations (5) and (6) to pH 5.5 and 8.0, typical of sweat secretion by eccrine glands and in large skin folds, respectively.

The theoretical and experimental response curves for the Na^+^-selective sensors were found to be in good agreement (see Figure 1 and Figure S2A–E). The calculated and experimental response medians, as well as ion-exchange/coextraction constants (see Table S4), showed insignificant differences, except for composition **8**, where the discrepancy between the theoretical and observed response median reached 0.3 log units.

In the case of chloride-selective optodes, the expected and observed coextraction constants did not converge when using the complexation constant from the literature. The response range for compositions **10–12** is shifted toward lower NaCl concentrations compared to the predicted range. This discrepancy may be attributed to inaccuracies in the literature data regarding the complexation of the ionophore with the chloride anion. Based on the experimental data, the ion–ionophore complexation constant was re-estimated using Equation (6), giving log*β_XL_*(Cl II) = 4.17 ± 0.07. This value is approximately 0.6 log units higher than that reported in [44]. The newly determined constant was further used to recalculate the response of the chloride-selective sensors and provided decent agreement between the calculation and experiment (Figure 1B and Figure S2F–H) except for composition **14**, where the discrepancy in the inflection point was about 0.5 log units. This may be due to the use of a different batch of chloride ionophore II, which had been stored longer and had partially degraded. Furthermore, the generally recognized theory expressed by Equation (6) successfully predicted the response median but failed to predict the response slope in the case of composition **12**, which appears to require separate investigation.

Correlations were observed between the composition of the sensing phase and the response range and sensitivity of the resulting optodes (Table S4). An increase in ionophore content in Na^+^-selective optodes led to a slight loss in sensitivity. A decrease by factors of 1.1 and 1.3 was observed when comparing responses of the compositions **2** and **3** (ionophore Na X), and **5** and **8** (ionophore Na VI), respectively. A similar decrease in sensitivity with increasing ionophore content was noticed for Cl^−^-selective optodes (compositions **10**, **11**). However, in this case, the loss of sensitivity was accompanied by an expansion of the response range from 2.6 to 3.5 log units.

Among Na^+^-sensors, those containing the less lipophilic cation exchanger T*p*ClPB (composition **9**) demonstrated the highest sensitivity. No significant change in sensitivity was observed when Ch I (composition **1**) was replaced with Ch III (composition **2**).

The width of the response range for all Na^+^-selective optodes was within 2.5–2.8 log units. For Cl^−^-selective sensors, composition **12** with a large excess of ionophore over chromoionophore exhibited a narrow response range (0.9 log units) and the greatest sensitivity. In contrast, composition **14,** with a much lower ionophore-to-chromoionophore molar ratio, delivered a significantly broader response range (3.3 log units).

The selectivity of the studied sensors was not independently re-assessed in this work. This methodological decision is based on the well-documented ionophore profiles reported in seminal literature and is justified by the physiological matrix under investigation, wherein NaCl overwhelmingly dominates over potential interferents, such as K^+^ or Ca^2+^.

The key criterion for selecting optimal compositions was a suitable response range enabling measurements in the physiological range of Na^+^ and Cl^−^ concentrations in sweat, ~5–100 mmol/L (approximately −2.3 to −1.0 in log units). Optodes based on chromoionophore II (pK_a_ = 10.2) demonstrated response ranges that fell far below (in the case of Na^+^-selective sensors) or above (for Cl^−^-selective optodes) the required concentration ranges and were therefore not further studied.

The Na^+^-selective compositions **2**–**4** showed an optimal optical response in the desired concentration range at an intermediate pH of 6.5 (see Figure 1 and Figure S2), whereas composition **1**, based on the calculated response, can be used to measure sodium concentrations in sweat samples with a pH of around 5.5. Compositions **5**, **8**, and **9** are suitable for Na^+^ detection in more alkaline sweat samples with a pH closer to 8. For measuring Cl^−^ anions in neutral sweat samples (pH 6.5), chloride compositions **11** and **14** can be chosen. Compositions **10** and **12** demonstrated sufficiently suitable response medians for determining chloride anions in the range of interest at pH 5.5 and 8, respectively. It is important to note that the theoretical extrapolations for pH 5.5 and 8.0 presented in Figure 1 are not purely predictive. The validity of these calculations is directly corroborated by the experimental performance of the selected formulations, as evidenced by hysteresis measurements at extreme pH values discussed below and presented in Figure S4. Specifically, the practical response data for compositions 1 and 10 (optimized for pH 5.5) alongside compositions 5 and 12 (optimized for pH 8.0) confirm that these theoretical models accurately reflect real sensor operating range suitable for measurements at the extreme pH limits of human sweat.

As shown by the responses of compositions **1** and **2**, replacing the Ch I (pK_a_ = 12) with less acidic Ch III (pK_a_ = 13.4) leads to a shift of the response median toward more concentrated solutions by approximately 1.3 log units, which is close to ΔpK_a_ (Figure S3A). This observation corresponds to well-established formalism [39]. Figure S3B shows the effect of substituting the ionophore in otherwise identical compositions **2** and **5**. The nearly 1.0 log unit shift of the response range coincides with the logarithmic difference in complex formation constants of sodium ionophores Na X and Na VI. Finally, changing the quantitative composition, namely, increasing the ionophore content, leads to the expected effect of decreasing the measurable concentration (compare the plots for compositions **2** and **3** in Figure S3C) [38]. The behaviors of compositions 3 and 4 were virtually indistinguishable, resulting in coinciding calibration curves. As described by Equation (5), the change in chromoionophore (pK_a_) and stoichiometry was precisely compensated by the distinct stability constant (logβ_XL_) of the ionophore. Therefore, only composition 3 was retained for further study, while composition 4—being its functional analog—was omitted from subsequent experiments and the Supplementary Material.

**Figure 2:**
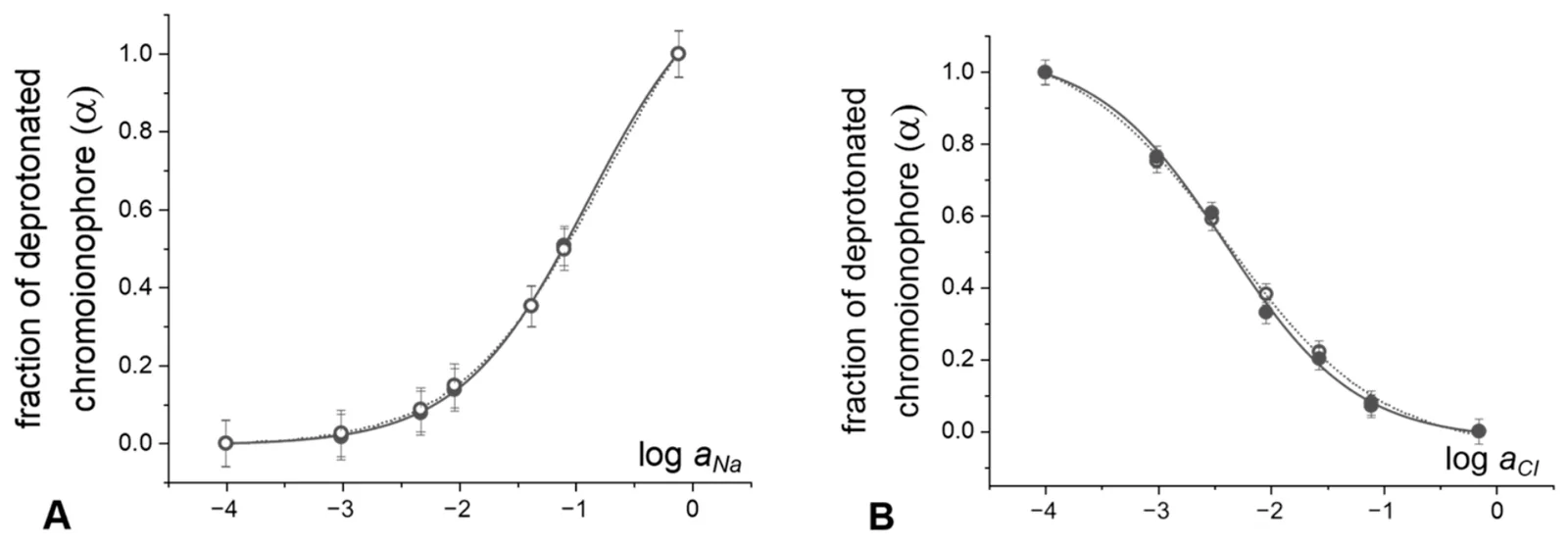
Hysteresis of the optical response for compositions **2** (**A**) and **11** (**B**), estimated spectrophotometrically, pH 6.5. Solid symbols—increasing NaCl concentration; open symbols—decreasing NaCl concentration. Lines—Boltzmann function fitting (Equation (3)). The error bars represent indirect measurement error estimated from photometric repeatability.

The hysteresis of the optode response was estimated (i) spectrophotometrically for the spin-coated optode films of compositions **1** and **10** suitable for acidic sweat samples, **2, 3, 11** and **14** for neutral sweat, and **5** and **12** for alkaline samples; (ii) by means of macrophotography followed by DCA for the optodes of compositions **2, 3, 14** fabricated in the form of drop-cast arrays. The results are shown in Figure 2 for compositions **2** and **11**, and in Figure S4 for the remaining compositions.

The tested compositions generally showed no significant hysteresis, with the maximum difference in analyte activity measured at α = 0.5 on the ascending and descending branches, not exceeding 0.09 log units in most cases. However, composition 14 was a notable exception, exhibiting a discrepancy of 0.5–0.8 log units for both registration methods. This can again be explained by partial degradation of the used batch of ionophore. To mitigate these performance failures during field deployment, the chemical integrity of the ionophore must be verified prior to membrane fabrication. This can be achieved through routine quality control protocols, such as thin-layer chromatography (TLC) or UV-Vis spectrophotometry. Moving forward, we plan to conduct a systematic stability study of pristine ionophores across various storage environments.

**Figure 3:**
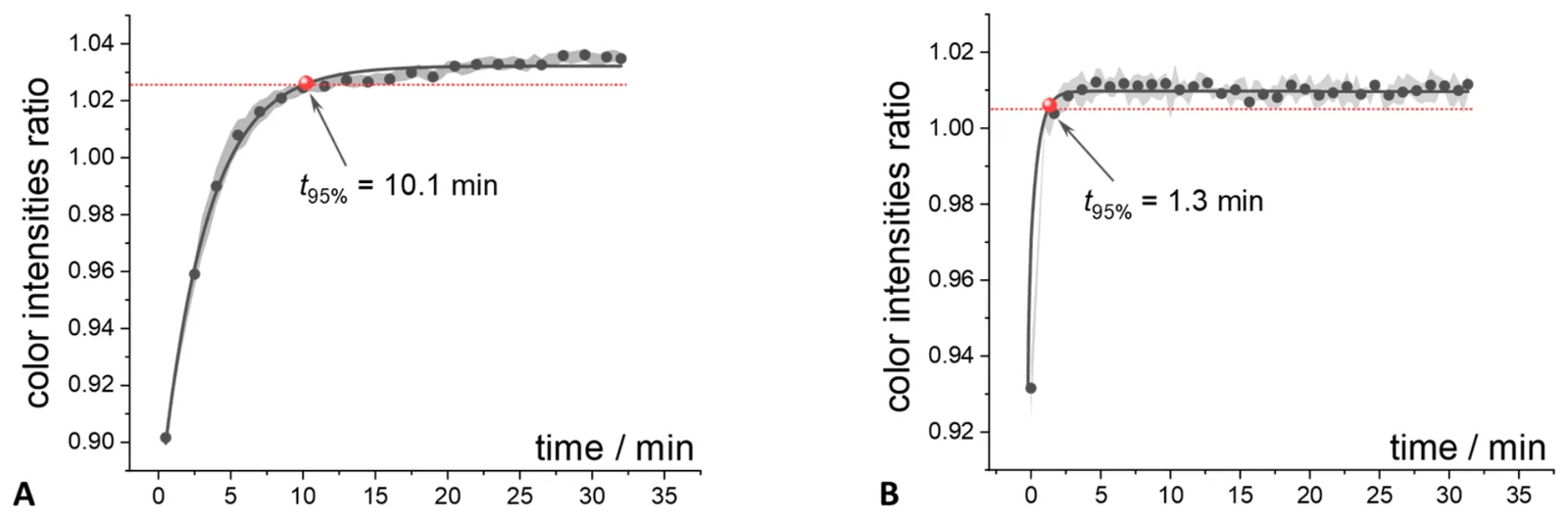
Dynamic response curves for drop-cast optodes on a Teflon substrate upon an increase in NaCl concentration from 10^−2^ to 10^−1^ mol/L at pH 6.5 (composition **2**). (**A**)—membrane prepared from the THF solution with well-established optode-to-THF volume ratio of 1:8; (**B**)—optode obtained from the THF solution with reduced optode-to-THF volume ratio (1:15). Shaded areas indicate the standard deviation (n = 8).

While the response time for thin spin-coated films (1.5–3 μm thick) was on the order of seconds, thicker drop-cast optodes (15–20 μm) exhibited significantly slower kinetics, with response times reaching 10–15 min (see Figure 3A and further examples in Figure S11 for artificial sweat measurements). In the case of drop casting, the highly hydrophobic Teflon surface impedes the spreading of the optode droplets, thereby limiting their distribution over a larger area.

It is well established that the response time depends quadratically on the membrane thickness [39]:

(7) $$t_{95\%}=1.13\frac{d^{2}}{D_{m}}$$

where d denotes the membrane thickness, and $D_{m}$ stands for the diffusion coefficient within the membrane phase (10^−8^ cm^2^/s [39], which is typical for PVC-DOS membranes with the polymer-to-plasticizer ratio used in our study). Consequently, to accelerate sensor equilibration, it is necessary to reduce the optode thickness. To achieve this, the initial optode cocktail was diluted approximately two-fold with THF (using a volume ratio of 1:15 instead of the standard 1:8). With a typical optode-to-THF volume ratio of 1:8, the dry membrane thickness, estimated from the response time using Equation (7), was approximately 23 μm. However, due to the reduced amount of solid components per unit volume of THF and the altered surface tension of the optode solution, the final membrane thickness decreased and was estimated to be 8.5 μm. This reduction accelerated the response by nearly 8-fold (Figure 3B). The optimized protocol was further employed to prepare optode compositions containing TiO_2_.

### 3.2. Optimization of Measurement Protocols

The simplification of signal registration systems is often accompanied by a degradation in sensor analytical performance if not properly designed. To evaluate the quality of readouts when replacing bulky spectrophotometry with digital colorimetry, we adopted the approach proposed by the authors of ref. [32] who proposed the parameter robustness (Rob). This metric allows for a quantitative assessment of major factors that may vary depending on the experimental protocol and unambiguously determines the advantages of a particular signal registration method. In this work, robustness is evaluated as the ratio of the dynamic range to the geometric mean of the standard deviations for each sensor across the entire concentration range (see Equation (4)). The higher this parameter, the superior the measurement quality. In this section, drop-cast optode arrays were exclusively employed to verify whether conventional thin-film spectrophotometry can be substituted by optode array imaging combined with DCA.

To assess the robustness of the optodes under various signal registration conditions, two compositions differing in the nature and quantities of active components (compositions **1** and **12**) were selected. Arrays containing nine individual sensors were calibrated in NaCl solutions (6–8 concentrations) at pH 5.5 and 8 for compositions **1** and **12**, respectively. The robustness analysis covered three key parameters: the type of image acquisition device, the illumination source and conditions, and the shooting angle. The capturing devices included a research-grade microscope camera, a consumer-grade camera, and two smartphones of different price categories. Illumination was varied using incandescent lamps (at fixed and randomly changing angles), the built-in flash of **smartphone 2**, and ambient room light (office LED lamps). For all setups, images were acquired using **smartphone 2**. The shooting angle was varied from normal to the Teflon surface to 45°, as well as dynamically within the range from 45° to 90°.

**Figure 4:**
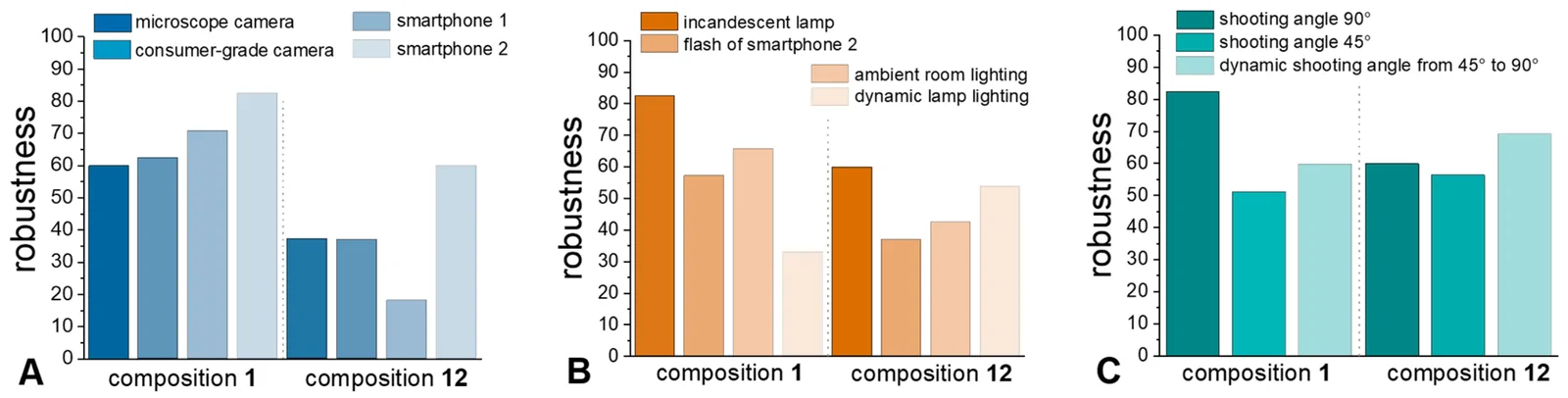
A comparison of robustness values for different measurement protocols: (**A**)—various recording devices under fixed incandescent lamp illumination; (**B**)—various light sources, axial shooting with **smartphone 2**; (**C**)—capturing with **smartphone 2** with built-in flash at different shooting angles.

For comparison, Figure S5 presents the normalized irradiation spectra of the aforementioned light sources alongside the typical absorption spectrum of the chromoionophore. The obtained values of robustness are presented in Figure 4.

The robustness values obtained using a research-grade camera and a consumer camera were comparable for both optode types; notably, the higher-quality smartphone outperformed both mentioned cameras in terms of robustness (Figure 4A). Overall, the results demonstrate that the use of simple and accessible devices, such as smartphones, for optical signal registration does not lead to a critical decrease in robustness compared to laboratory instruments. For both optode compositions, all protocols yielded comparable results (except for **smartphone 1** with composition **1**), while **smartphone 2** consistently exhibited the highest robustness value.

Since the analytical signal is derived from a photograph of the sensor array, external illumination is the primary factor that can directly affect the measurements. Figure 4B shows a significant role of the light source; in our experiment, incandescent lamps provided the highest robustness values (Figure 4B), even under dynamic illumination mode (for the case of composition **12**). Although the smartphone flash features a high-quality white emission spectrum (Figure S5), it generally delivered lower robustness values compared to incandescent lamps. This can be attributed to the greater discreteness and spectral inhomogeneity of LED emission relative to thermal sources. The relative improvement in robustness observed under ambient room light, which, in fact, shares a similar emission spectrum with the smartphone’s white LEDs (Figure S5), can be explained by the superior homogeneity of illumination over the area of interest. This effect arises from indirect positioning of the light source and scattering within the environment, which prevents the formation of local shadows.

Based on Figure 4C, it can be concluded that the positioning of the recording device has a rather weak influence on the reliability of the results. However, to maintain its impact constant under real measurement conditions, it is advisable to keep the capturing angle fixed and preferably close to 90° relative to the substrate surface.

In real-world applications, the intrinsic coloration of a sample can interfere with optical measurements performed using transparent PVC-based optode films. To mitigate this background contribution, we adopted the strategy described in ref. [33], which involves incorporating light-scattering microparticles into the polymeric matrix to render the optode opaque.

To optimize the density and homogeneity of the dispersion within the polymeric phase, several protocols for incorporating nanosized TiO_2_ particles were evaluated. The mass ratio of PVC to TiO_2_ was varied from 1:0.25 to 1:1. Particle dispersion was achieved using either an ultrasonic bath or vortex mixing, while the duration of treatment was also systematically varied. Then, the resulting compositions were deposited on flexible polypropylene supports to form an array and imaged against a black background.

The dependences of the mean grey intensity of chromoionophore-free PVC films on the TiO_2_ content, dispersing method, and sonication time are presented in Figure S6, while Figure S7A shows representative optical images of the resulting TiO_2_-containing films placed on various colored backgrounds. Overall, introducing TiO_2_ particles to the membrane effectively mitigates the background color interference. Under the experimental conditions used (black background), the average grey intensity, by its proximity to pure white, reflects the overall opacity of the film. Furthermore, the standard deviation of the pixel intensities within a single array element reflects the degree of TiO_2_ heterogeneity in the bulk polymer.

Figure S6A suggests that, for ultrasonication, a processing time of 60 min is sufficient to achieve maximal background filtering; longer exposure does not improve opacity and leads to less uniform distribution of TiO_2_ throughout the film bulk due to particle agglomeration. As expected, increasing the TiO_2_ fraction results in higher film opacity and more efficient suppression of the background signal (Figure S6B). Generally, vortex dispersion (Figure S6C) outperforms ultrasonication, yielding higher mean values of grey and producing a more homogeneous spatial distribution of the dopant. These characteristics further improve with prolonged mixing times. Therefore, a protocol comprising 20 min of vortexing at a PVC:TiO_2_ mass ratio of 1:1 was established as optimal for incorporating the color-filtering agent into the optode membranes.

**Figure 5:**
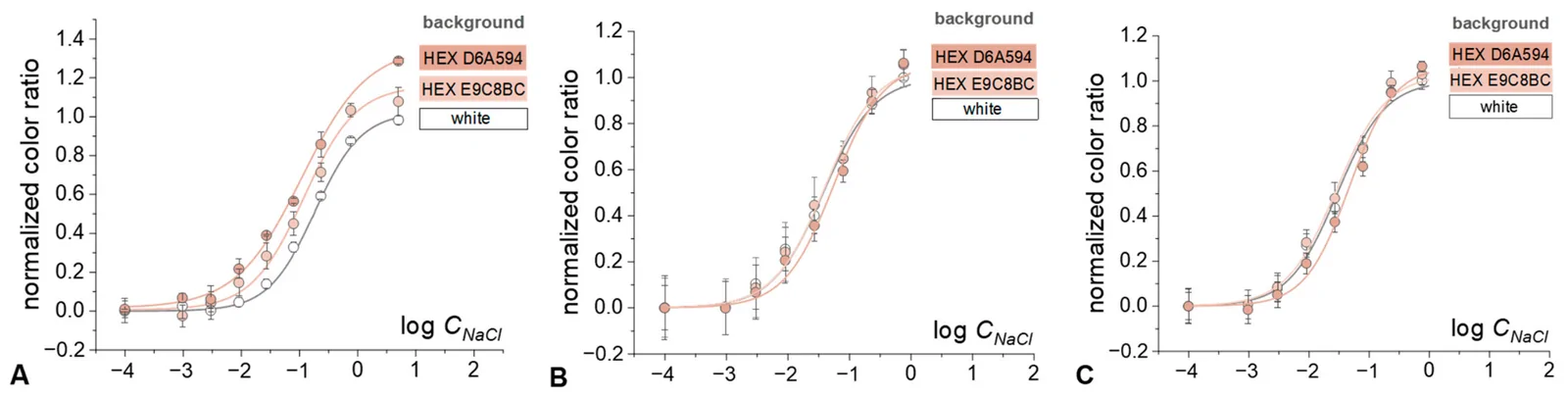
The response of optical sensors of compositions **2** ((**A**), no TiO_2_) and **15** (**B**,**C**) on various backgrounds, pH = 6.5; (**B**)—dispersing via ultrasonic bath (60 min); (**C**)—dispersing via vortex (20 min). Tested on white, HEX E9C8BC, and HEX D6A594 backgrounds (indicated in the plots). Symbols—experimental data, lines—Boltzmann function fitting (Equation (3)), error bars—standard deviation (n = 5).

Figure 5 and Figure S7B compare the colorimetric responses of drop-cast optode arrays prepared with and without titanium dioxide (compositions **15** and **2**, respectively) when measured against various colored backgrounds simulating skin hues. The arrays were cast onto a transparent polypropylene support, and the optical signals were captured using a digital microscope camera coupled with an incandescent illumination system.

Figure S7B unambiguously demonstrates an expansion of the optical response span upon incorporation of the light-scattering component. This observation is consistent with established optical principles: the introduction of scattering particles increases the effective path length of light within the membrane due to multiple internal reflections, which in turn amplifies the magnitude of the colorimetric change [33].

Meanwhile, the plots in Figure 5A demonstrate a trend of increasing response amplitude for optical sensors without TiO_2_ as the background color shifts from white to beige and dark beige. This artifact arises because transparent TiO_2_-free sensors do not mask the underlying substrate; since the chosen backgrounds exhibit high reflectance in the red spectral region, they elevate the red-to-green ratio captured by the camera. The influence of this parasitic absorption becomes increasingly significant as the chromoionophore transitions into its deprotonated form.

In contrast, optodes doped with titanium dioxide at a 1:1 ratio with PVC exhibit a stable response regardless of the background hue. Nevertheless, a negligible residual trend persists, characterized by a minimal response span on a white background and a marginally expanded span against a dark beige background.

Finally, the effect of TiO_2_ addition on the response time of the optodes was studied using compositions **2** and **15** prepared from the diluted casting solutions (1:15 volume ratio of membrane components to THF). The dynamic response of the optodes with and without TiO_2_ to the change in NaCl concentration from 10^−4^ to 10^−1^ mol/L is shown in Figure S7C. Evidently, incorporating TiO_2_ into the polymeric phase at a 1:1 mass ratio does not significantly increase the response time.

To conclude, light-scattering agents such as TiO_2_ can be successfully implemented in polymeric optode membranes to render them opaque and effectively prevent interference from colored samples without compromising sensor sensitivity, response span, or measurement time.

### 3.3. Measurements in Artificial Sweat Solutions

The validation of the obtained optodes was conducted in model artificial sweat solutions (Table S1) across a physiological pH range of 5.5 to 8.0. Based on preliminary screening, optimal membrane compositions were selected: **1**, **2**, and **5** and **10**, **11**, and **12**, selective for sodium and chloride ions, respectively.

To ensure the composition of the artificial sweat solutions, acidic and alkaline samples were titrated potentiometrically using a glass pH electrode to determine the inherent buffer capacity (Figure S8A,B). The resulting buffer capacities covered pH ranges of 5.5–8.0 for acidic sweat and 7.0–9.1 for alkaline sweat. As the neutral sweat standard lacks an intrinsic buffer system, 0.01 mol/L HEPES buffer was added to maintain a stable pH of 6.5 within the target range of 6.0–8.0. In the neutral standard containing lactic acid, time-dependent precipitation of polylactides was observed. Consequently, optimal storage conditions were established (Figure S8C), and all subsequent solutions were stored at 4 °C in opaque beakers to prevent chemical degradation.

**Figure 6:**
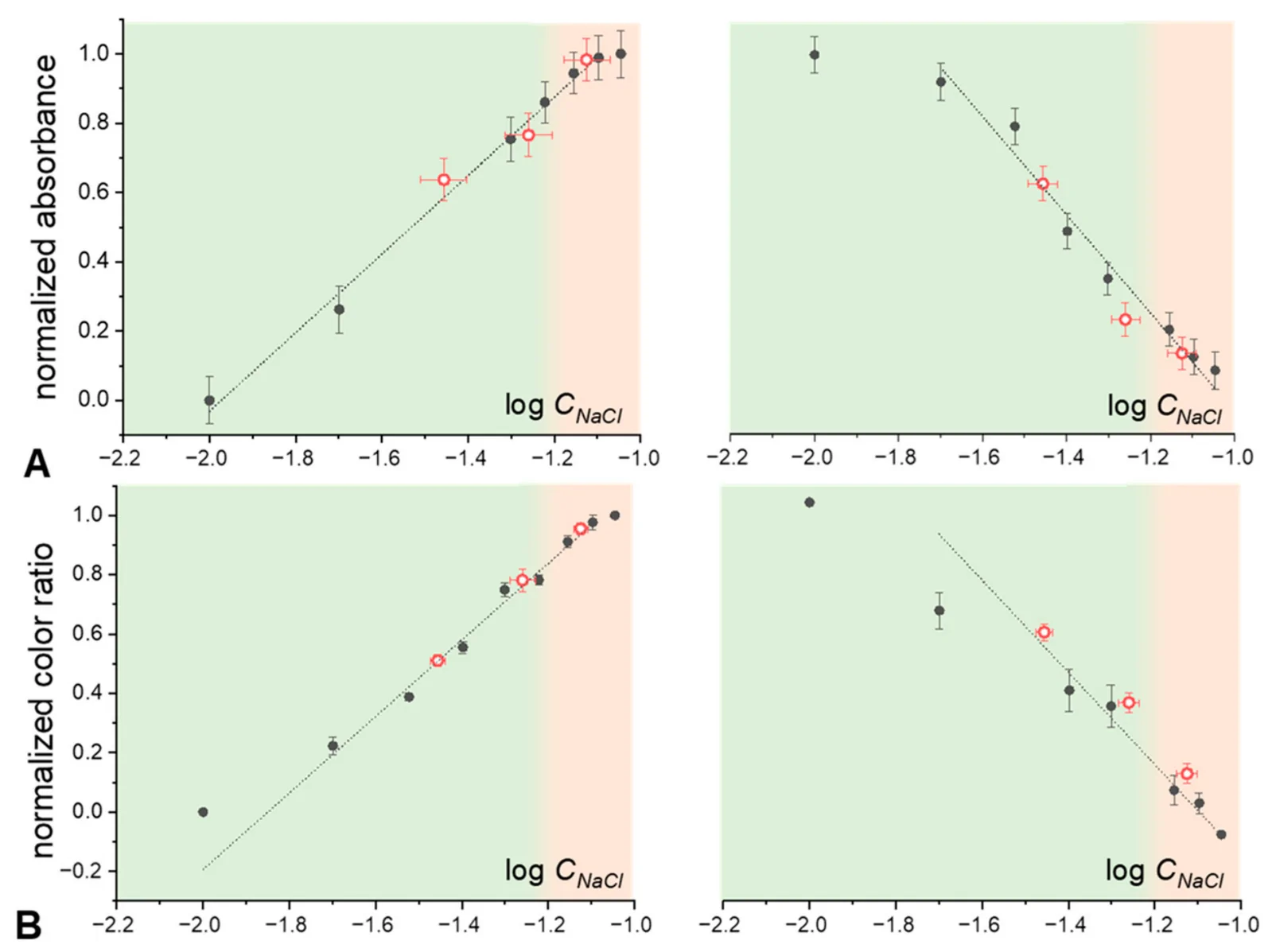
Calibration curves (solid symbols) for the sensors with compositions 2 (**left panels**) and 11 (**right panels**) in artificial sweat solutions at pH 6.5. (**A**)—spectrophotometric response of thin optode films; (**B**)—colorimetric response of drop-cast arrays acquired using a microscope camera. Open symbols—independently prepared artificial sweat samples used for recovery-based validation. Green shaded area—physiological concentration ranges of Na^+^ and Cl^−^ in sweat; orange shaded area—elevated concentrations. The error bars represent indirect measurement error estimated from photometric repeatability (**A**) or standard deviation of the experimental data (**B**, n = 6).

The response curves were recorded using two different recording devices: a spectrophotometer (for thin films) and a research-grade digital camera coupled with a microscope (for drop-cast arrays). For direct comparison, the data collected from the two methods were normalized to a unified scale. For spectrophotometry, the absorbance intensity of the protonated optode in contact with 0.1 mol/L HNO_3_ was defined as 1, while the signal in 0.1 mol/L KOH was defined as 0. For digital colorimetry, the red-to-green component ratio upon contact with 0.1 mol/L HNO_3_ was set to 1, and the ratio in 0.1 mol/L KOH was set to 0. Representative calibration plots obtained by these methods are presented in Figure 6 for compositions **2** and **11** and in Figure S9 for the remaining optodes.

**Figure 7:**
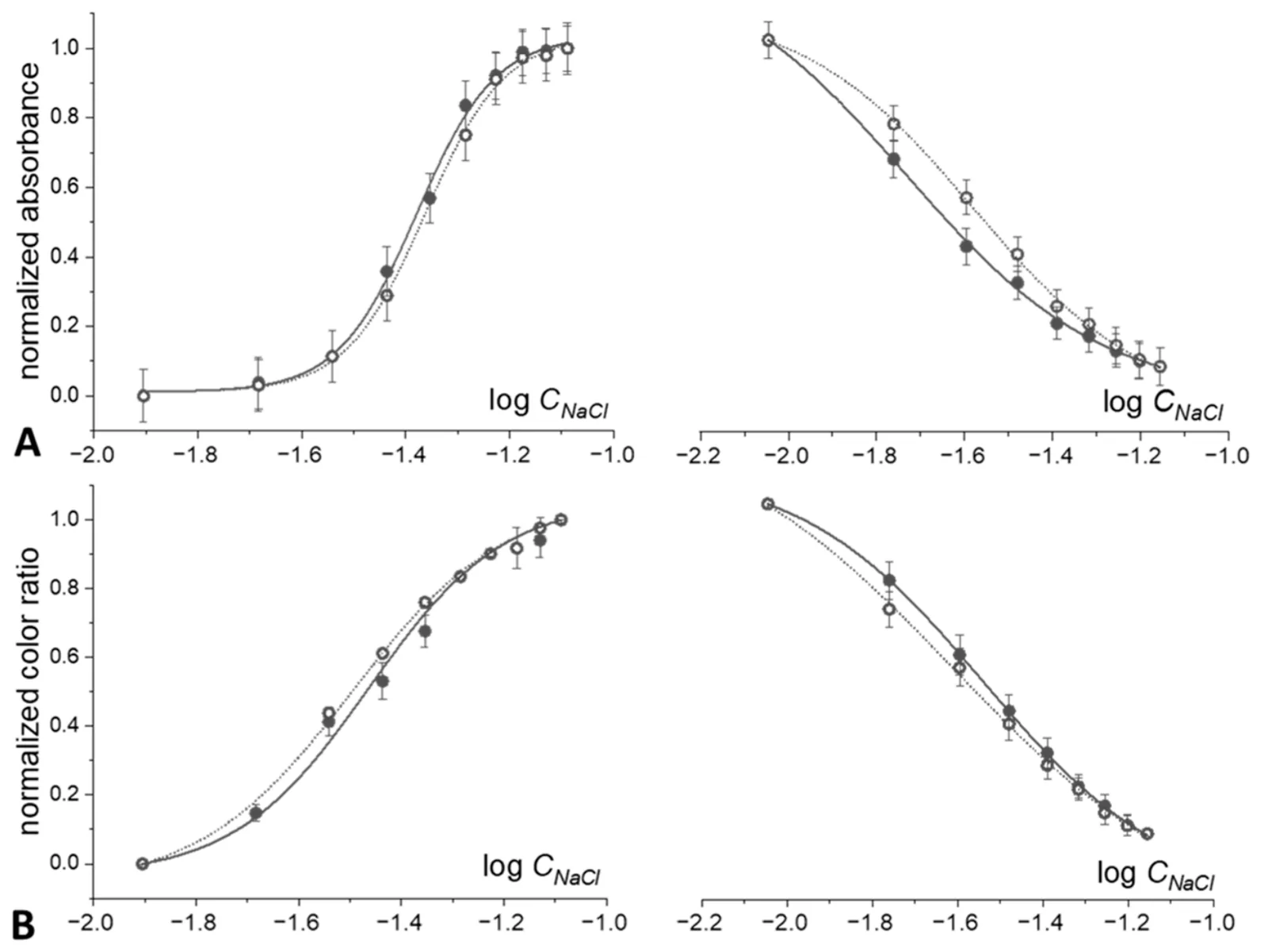
Hysteresis of the optical response of the sensors with compositions **1** (**left panels**, pH 5.5) and **12** (**right panels,** pH 8.0) in artificial sweat. (**A**)—spectrophotometric response of thin optode films; (**B**)—colorimetric response of drop-cast arrays acquired using a microscope camera. The error bars represent indirect measurement error estimated from photometric repeatability (**A**) or standard deviation of the experimental data (**B**, n = 6). Solid symbols—data recorded during stepwise increase in NaCl concentration; open symbols—the corresponding stepwise decrease. Lines—Boltzmann function fitting (Equation (3)).

To assess response hysteresis in artificial sweat, calibration curves obtained via spectrophotometry and macrophotography were compared for both increasing and decreasing analyte concentrations. Representative results obtained in an acidic sweat standard for compositions **1** and **12** are shown in Figure 7, while those for the remaining compositions are presented in Figure S10.

Both Na^+^- and Cl^−^-selective compositions showed good agreement between the ascending and descending branches of their respective calibration curves. However, hysteresis was more pronounced in the complex artificial sweat matrix than in pure aqueous NaCl solutions, particularly for chloride-selective sensors and in the case of using macrophotography for signal acquisition. This effect may be attributed to the thicker optode films in drop-cast arrays and incomplete equilibration with the artificial sweat solution in some cases.

The response time of the drop-cast sensors in artificial sweat samples was approximately 7.5 min for sodium-selective sensors and averaged 14 min for chloride-selective optodes (Figure S11). Nevertheless, rapid equilibrium is not essential for the intended epidermal application of these sensors, in which sweat composition is expected to be monitored over longer time intervals. Furthermore, if faster kinetics becomes necessary, the equilibration time may be reduced by diluting the membrane casting solution before deposition to produce thinner sensing films.

To prepare model artificial sweat samples for the recovery-based validation (Table S2), calibration solutions were mixed in different proportions to obtain samples with known Na^+^ and Cl^−^ concentrations. Calibration curves for thin optode films and drop-cast sensor arrays were recorded using spectrophotometry and macrophotography combined with DCA, respectively. The concentrations of the validation samples were then back-calculated from the linear domains of the corresponding calibration curves. The results are presented in Figure 6 and Figure S9 and summarized in Table S5. Both tested measurement approaches yielded comparable quantitative results for both ions. The deviation between the found and target concentrations did not exceed 10% on a logarithmic concentration scale, corresponding to a minimum recovery of 93.9%. These results support the potential applicability of the developed sensors for Na^+^ and Cl^−^ determination in sweat. Furthermore, given its portability and simpler instrumentation, macrophotography may provide a practical alternative to conventional spectrophotometry for in situ analysis with optical sensor arrays.

**Figure 8:**
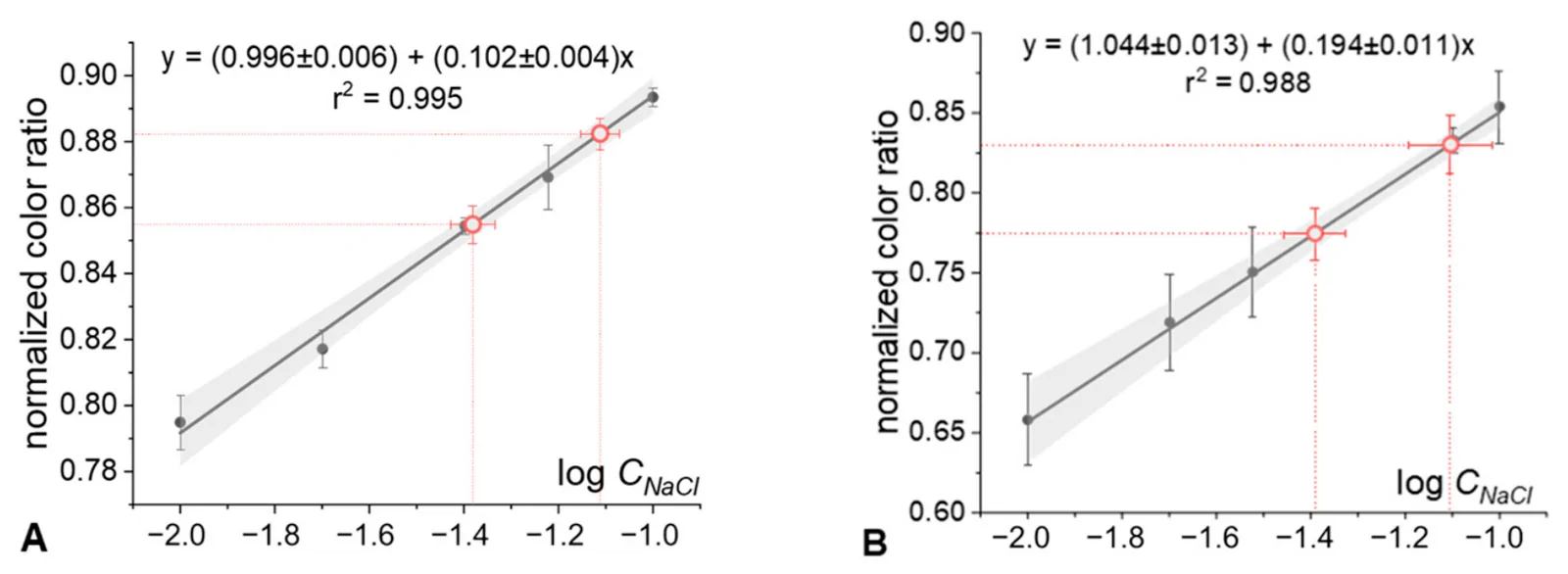
Calibration curves (solid symbols) of the composition **4** sensor array under simulated epidermal measurement conditions at pH 6.5, obtained by macrophotography followed by DCA: (**A**)—using a microscope camera under incandescent lamp illumination; (**B**)—using a smartphone camera (**smartphone 3**) with built-in LED flash. The parameters of linear approximation are indicated in the plots. Open symbols—measurements in artificial sweat samples used for validation. The error bars represent standard deviation of the experimental data (n = 6). Shaded areas indicate confidence intervals (p = 0.95).

To evaluate sensor performance under conditions more closely resembling epidermal measurements, artificial sweat solutions (pH 6.5) were absorbed onto filter paper strips to simulate a moist skin surface. Excess solution was removed by blotting, and the sensor array based on composition **4**, deposited on a flexible transparent polypropylene support, was placed in contact with the moistened paper and allowed to equilibrate. Calibration curves were acquired using a microscope camera under incandescent lamp illumination (Figure 8A) and **smartphone 3** with built-in LED flash (Figure 8B). The resulting calibration curves were then used to determine Na^+^ concentrations in two artificial sweat validation samples, with the results summarized in Table 1.

Under both imaging conditions, the normalized color ratio showed an approximately linear dependence on the logarithm of NaCl concentration within the studied range, with R^2^ values of 0.995 and 0.988 for the microscope and smartphone cameras, respectively. Measurements obtained using the microscope camera exhibited lower variability than those acquired using the smartphone, particularly at the higher concentration level. Nevertheless, the two imaging approaches produced comparable mean recoveries, ranging from 84 to 86% for the microscope camera and from 82 to 88% for the smartphone (Table 1). The smartphone measurements showed greater uncertainty, especially for the 90 mmol/L sample, but the built-in LED flash provided sufficiently reproducible illumination to distinguish between the tested concentration levels. Overall, the experiment demonstrates the feasibility of acquiring the optode response from a flexible sensor array under conditions simulating contact with a moist epidermal surface. However, the recoveries below 90% indicate a negative analytical bias that should be considered and further minimized before application to real sweat samples. This drop in recovery from 93% in bulk testing to 82–88% in the epidermal simulation is mainly due to the different calibration modes used. For the bulk measurements, we accounted for the complex background matrix by evaluating individual ionic activities through the Debye–Huckel approximation. In contrast, the epidermal simulation was calibrated against the nominal NaCl concentration. Since activity coefficients decrease as ionic strength grows, the optodes sense an effective activity that is lower than the nominal concentration, which inherently causes a negative analytical bias. Furthermore, the signal was likely reduced by incomplete thermodynamic equilibration in the thin fluid film, along with optical scattering from the skin-like surface. To resolve these issues in the upcoming Part 2 on a wearable device study, three targeted optimizations will be implemented: (1) adopting a matrix-matched, activity-based calibration to eliminate the ionic strength bias; (2) integrating a micro-volume sweat collection interface for better analyte equilibration; and (3) enhancing optical isolation to minimize background scattering.

## 4. Conclusions

The conducted systematic screening identified specific Na^+^- and Cl^−^-selective optode compositions capable of operating reliably across the full spectrum of physiologically relevant sweat pH values at normal and elevated concentrations of sodium and chloride ions.

This work establishes smartphone macrophotography coupled with digital color analysis as a highly accessible and practical alternative to laboratory spectrophotometry for in situ measurements. High-end mobile devices provided superior robustness compared to consumer cameras due to their advanced image processing algorithms and consistent LED flash spectra. This finding significantly lowers the barrier for translating these sensors into portable diagnostic tools.

It has been experimentally demonstrated that introducing light-scattering particles into the polymeric sensor is an effective strategy for mitigating parasitic absorption caused by inherent sample color, thereby ensuring stable analytical signals regardless of the underlying substrate hue.

While developed specifically for the quantitative determination of electrolytes in the context of diagnosing cystic fibrosis, the investigated sensing systems possess broader applicability. Beyond analysis of body fluids, the studied colorimetric sensors, characterized by tunable response range, sufficient sensitivity, low hysteresis, and compatibility with inexpensive readout hardware, are well suited for solving diverse analytical tasks: environmental monitoring of salinity in water bodies, quality control of food products, and industrial process monitoring where miniaturization and portability are prioritized over absolute spectroscopic precision.

## Figures and Tables

**Table 1:** Determination of Na^+^ in artificial sweat validation samples under simulated epidermal measurement conditions.

C_target_, mmol/L	Microscope Camera		Smartphone 3	
	C_found_, mmol/L	Recovery, %	C_found_, mmol/L	Recovery, %
50	42 ± 5	84 ± 10	41 ± 6	82 ± 12
90	77 ± 7	86 ± 8	79 ± 16	88 ± 15

## Data Availability

The raw data supporting the conclusions of this article will be made available by the authors on request.

## Supplementary Materials

The following supporting information can be downloaded at https://www.mdpi.com/article/10.3390/s26185774/s1, Figures S1–S11: absorption spectra of the studied optodes; spectrophotometric and colorimetric response of the optodes and its hysteresis in pure solutions and artificial sweat samples; comparison of the response of Na^+^-optodes of different composition; irradiation spectra of the light sources used in the study; the mean grey intensity dependences, optical images and characteristics for TiO_2_-containing PVC films; potentiometric titration of acidic and alkaline model sweat and stability of pH of the neutral sweat standard; the dynamic response curves for the drop-cast sensors measured in artificial sweat; Tables S1–S5: composition of artificial sweat solutions; compositions of the fabricated optodes; analytical characteristics of the studied optodes; the results of recovery studies.
