# Supplementary material for: Tailoring Na+ and Cl−-Selective Colorimetric Optode Arrays for Wearable Sweat Analysis: Composition Optimization and Measurement Conditions
Source: Sensors (Basel). 2026 Sep 11;26(18):5774. doi: 10.3390/s26185774 (PMC13611156; doi:10.3390/s26185774)
Supplement: Supplementary file 1 [file sensors-26-05774-s001.zip › sensors-4505589-supplementary.pdf]

Tailoring Na^+ and Cl^--Selective Colorimetric Optode Arrays for Wearable Sweat Analysis: Composition Optimization and Measurement Conditions

Vasiliy S. Syutkin[1], Ivan P. Gryazev[1], Daria A. Chetverikova[1], Andrey V. Kalinichev[2], and Maria A. Peshkova[1]*

[1]Institute of Chemistry, Saint Petersburg State University
7/9 Universitetskaya Emb., 199034, St. Petersburg, Russia

[2]Aarhus University, Department of Biology — Microbiology
Ny Munkegade 114, 8000 Aarhus C, Denmark

[*]corresponding author: m.peshkova@spbu.ru

SUPPLEMENTARY MATERIAL

Table S1. Artificial sweat solutions (background components). The concentrations are given in g/L.

**component**	neutral sweat standard [1]	"alkaline" sweat standard [2]	"acidic" sweat standard [2]
pH	6.50	8.00	5.50
lactic acid (88%)	1.20	–	–
urea	1.30	0.50	–
tris(hydroxymethyl)aminomethane (Tris)	–	5.00	–
nitrilotriacetic acid	–	0.50	–
L-histidine $HCl \cdot H_2O$	–	–	0.50
$NaH_2PO_4 \cdot 2H_2O$	–	–	2.20

Table S2. NaCl concentration in the prepared artificial sweat solutions.

	**NaCl concentration, mmol/L**									
"alkaline" sweat standard	10	20	30	40	50	60	70	80	90	–
"acidic" sweat standard	14	24	34	44	54	64	74	84	94	104
neutral standard	10	20	30	40	50	60	70	80	90	–

Table S3. Compositions of the fabricated optodes.

#	matrix (mass ratio)	active components C : R : L	concentrations C : R : L (mmol/kg)	operational pH
	**sodium-selective compositions**			
**1**	PVC : DOS (1 : 2.75)	Ch I : NaHFPB : Na X	10 : 11 : 60	pH 5.5
**2**		Ch III : NaHFPB : Na X	10 : 11 : 60	pH 6.5
**3**		Ch III : NaHFPB : Na X	10 : 11 : 100	pH 6.5
**4**		Ch I : NaHFPB : Na VI	10 : 11 : 60	pH 6.5
**5**		Ch III : NaHFPB : Na VI	10 : 11 : 60	pH 8.0
**6**	PVC : DOS (1 : 2)	Ch II : NaHFPB : Na VI	10 : 11 : 60	not suitable
**7**		Ch II : NaHFPB : Na VI	20 : 35 : 40	not suitable
**8**		Ch III : NaHFPB : Na VI	10 : 11 : 100	pH 8.0
**9**		Ch III : KTpClPB : Na VI	10 : 11 : 80	pH 8.0
	**chloride-selective compositions**			
**10**	PVC : DOS (1 : 2.75)	Ch I : Cl II	10 : 21	pH 5.5
**11**		Ch I : Cl II	10 : 170	pH 6.5
**12**		Ch III : Cl II	10 : 105	pH 8.0
**13**	PVC : DOS (1 : 2)	Ch II : Cl II	30 : 40	not suitable
**14**		Ch III : Cl II	35 : 40	pH 6.5
**15**	PVC : DOS : TiO_2 (1 : 2 : X*)	Ch III : NaHFPB : Na X	10 : 11 : 60	pH 6.5

*X = 0.25, 0.5, 0.75, 1

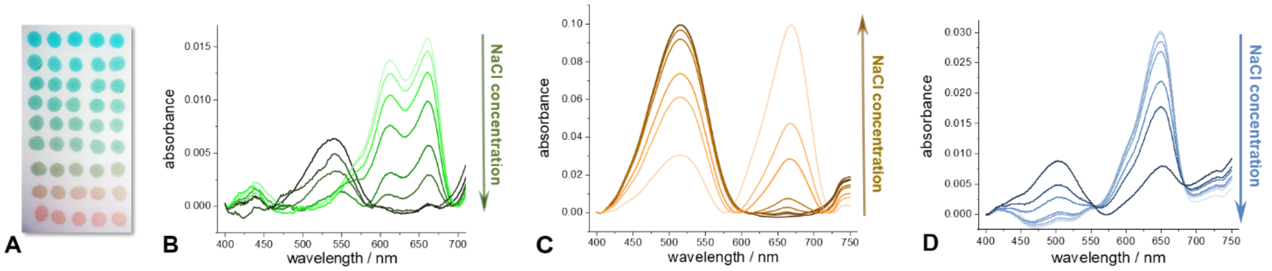

Fig. S1. A – an example of the obtained optode array (composition **2**), each row is equilibrated with the solution of different NaCl concentration (from 10^{-5} to 3 M), pH 6.5; B–D – absorption spectra for the optodes of compositions **1**, **13**, and **2** recorded NaCl solutions of different concentrations. Photometric accuracy ±0.002.

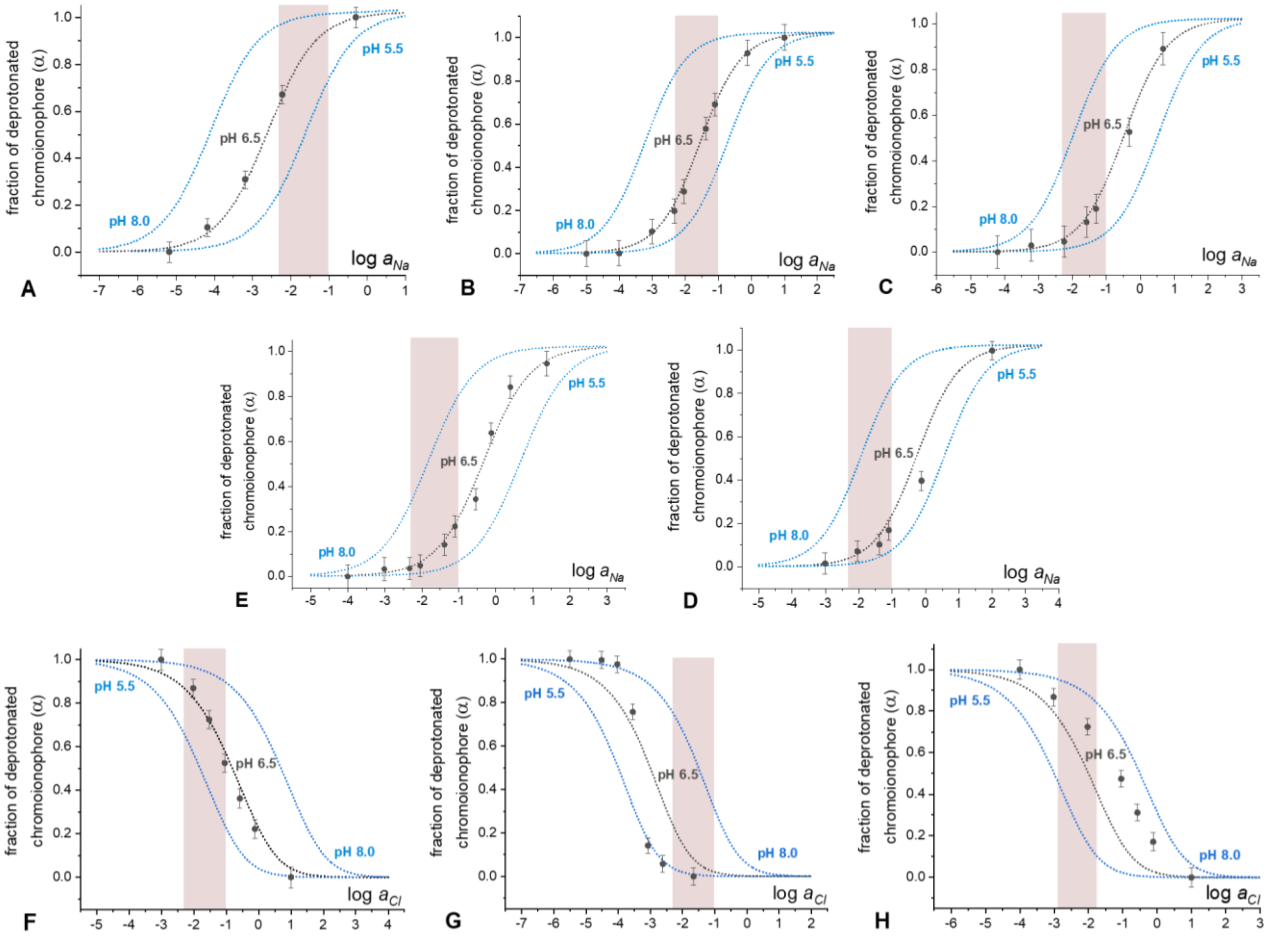

Fig. S2. The spectrophotometric response of the optodes of compositions: A – **1**; B – **3**; C – **5**; D – **8**; E – **9**; F – **10**; G – **12**; H – **14**, to sodium and chloride activities in the solution, respectively. pH = 6.5, symbols – experimental data, black lines – calculations with Eqs. 5 (A) and 6 (B) using the listed parameters and logβ_{XL}(Cl II) = 4.17. Blue lines – extrapolations of the curves computed at pH 6.5 to pH 5.5 and 8.0. Colored rectangles – the concentration range of interest. The error bars represent indirect measurement error estimated from photometric repeatability.

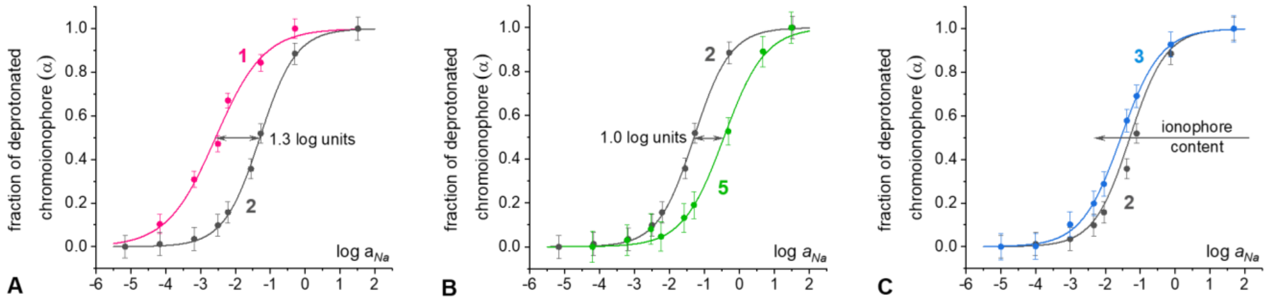

Fig. S3. Comparison of the response of Na^+-optodes of different composition: A – with different chromoionophores (composition **1** – Ch I, composition **2** – Ch III); B – with different ionophores (composition **2** – Na X, composition **8** – Na VI); C – with different ionophore concentration (composition **2** – 60 mmol/kg, composition **3** – 100 mmol/kg). pH = 6.5, symbols – experimental data, black lines – calculations with Eq. 5 The error bars represent indirect measurement error estimated from photometric repeatability.

Table S4. Analytical characteristics of the studied optodes and their calculated and experimental response medians and ion-exchange/coextraction constants.

composition	exchange/coextraction constant, log K_{exch}/log K_{coex}		response median, log a_0		response range, log units	sensitivity*, $(d\alpha/d\log a)_{a_0}$
	theory	experiment	theory	experiment		
**Na^+-selective optodes**						
**1**	−4.8	−4.9±0.9	−2.60	−2.57±0.06	2.7	0.46±0.04
**2**	−6.1	−6.2±0.7	−1.20	−1.277±0.021	2.8	0.475±0.016
**3**	−6.1	−6.3±0.7	−1.61	−1.55±0.03	2.6	0.421±0.021
**5**	−7.0	−6.9±0.6	−0.48	−0.43±0.05	2.8	0.355±0.022
**8**	−7.3	−7.4±0.6	−0.26	0.03±0.09	2.5	0.28±0.05
**9**	−7.3	−7.3±0.5	−0.33	−0.33±0.06	2.7	0.55±0.04
**Cl^--selective optodes**						
10	6.8	6.8±1.2	−0.62	−0.94±0.04	2.6	0.380±0.017
11	6.8	6.8±0.6	−1.60	−1.83±0.07	3.5	0.29±0.03
12	8.2	8.2±0.7	−2.78	−3.37±0.03	0.9	0.88±0.04
14	8.2	7.7±0.7	−1.78	−1.06±0.08	3.3	0.33±0.03

* estimated as a slope of the tangent in the inflection point of the response curve [3].

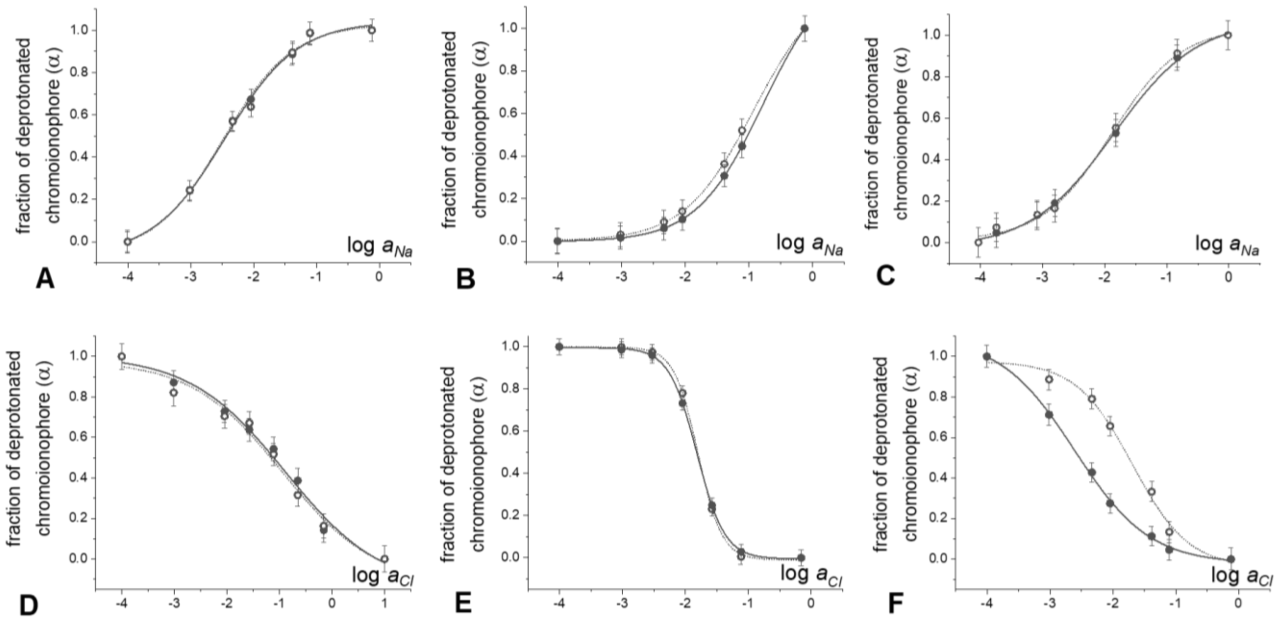

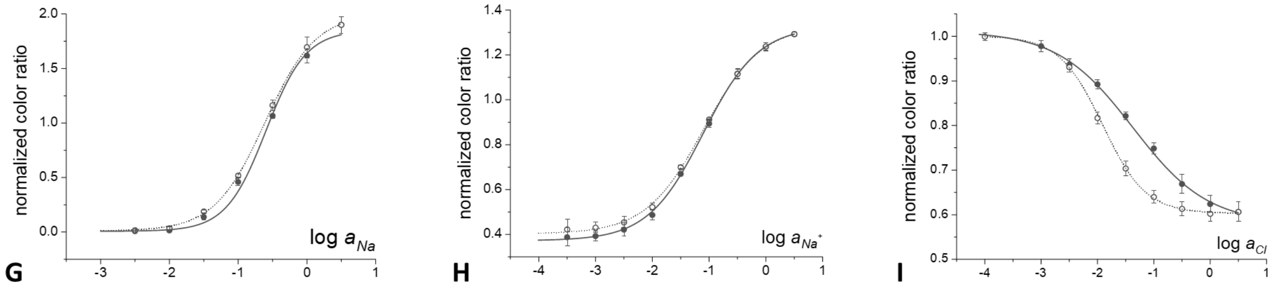

Fig. S4. Hysteresis of the optical response estimated spectrophotometrically (error bars – indirect measurement error estimated from photometric repeatability, A–F) and by macrophotography (error bars – standard deviation, n = 9, G–I). Solid symbols – increasing NaCl concentration, open symbols – decreasing NaCl concentration. Lines – Boltzmann function fitting (Eq. 3). A – composition **1**, pH 5.5; B – composition **3**, pH 6.5; C – composition **5**, pH 8.0; D – composition **10**, pH 5.5; E – composition **12**, pH 8.0; F – composition **14**, pH 6.5; G – composition **2**, pH 6.5; H – composition **3**, pH 6.5; I – composition **14**, pH 6.5.

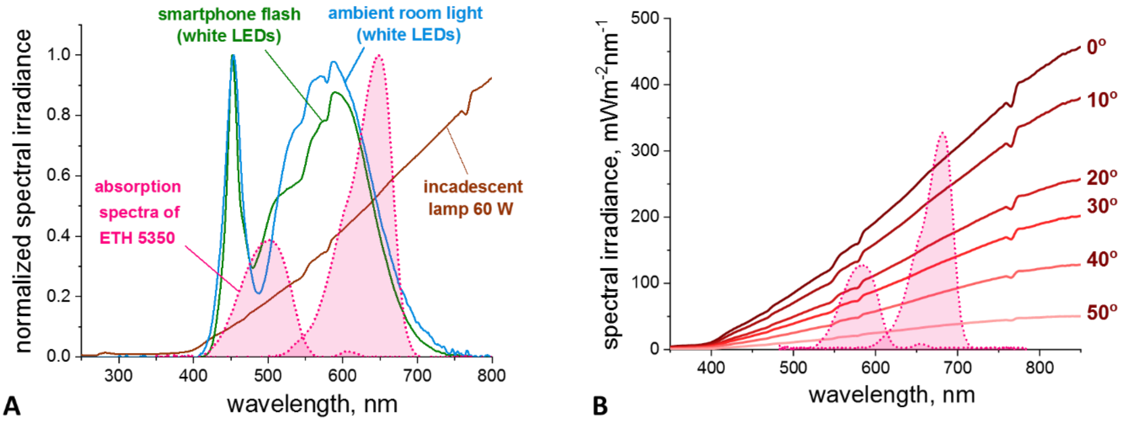

Fig. S5. A – normalized irradiation spectra of the light sources used in the study (indicated in the plot); B – irradiation spectra of the incandescent lamp at different measurement angles to the spectrometer. Filled area – absorption spectrum of Ch III (α = 0.5).

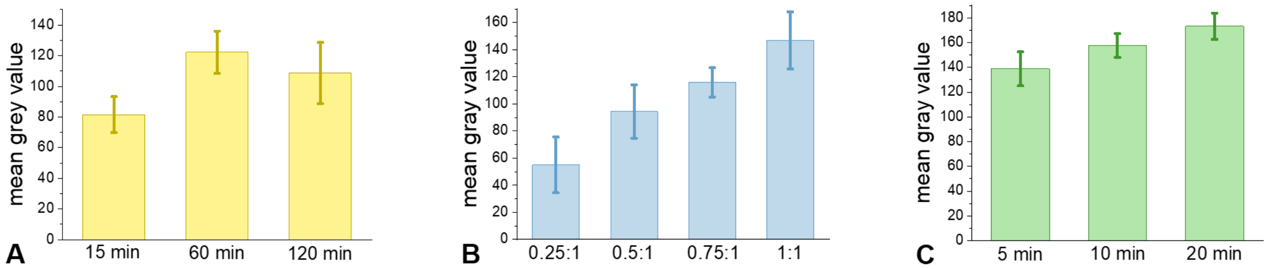

Fig. S6. The dependences of the average grey intensity for drop-cast chromoionophore-free plasticized PVC films deposited onto a transparent polypropylene substrate and photographed with a microscope camera against a black background (n = 5): A – on the TiO_2 ultrasonication time (shown in the plot, TiO_2:PVC = 0.5:1); B – on the TiO_2 to PVC mass ratio in the matrix (indicated in the plot; dispersion in ultrasonic bath for 60 minutes); C – on the centrifuging time in vortex (indicated in the plot; TiO_2:PVC = 1:1). Standard deviation was calculated from the mean (n = 5) value of SDs obtained from averaging grey intensities of individual pixels of the area of interest.

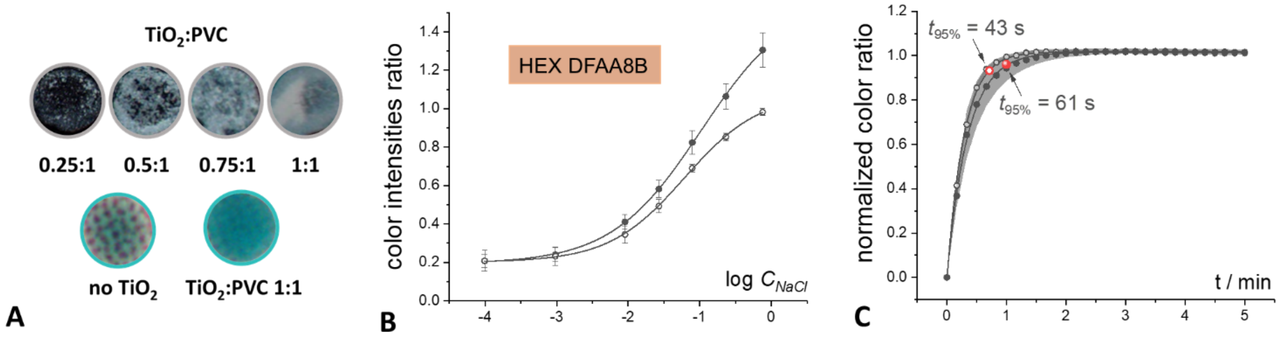

Fig. S7. A – optical images of the resulting TiO_2-containing drop-cast films placed onto various colored backgrounds: black (upper row), ornamented red and white paper (lower row); B – the response curves obtained from TiO_2-free (open symbols) and TiO_2-containing (solid symbols) optode arrays against HEX DFAA8B background (composition **15**, pH 6.5). Symbols – experimental data (error bars represent standard deviation, n = 5), lines – Boltzmann function fitting (Eq. 3); C – the response time of the drop-cast sensors with (solid symbols) and without (open symbols) TiO_2 in the sensor matrix (composition **15**, pH 6.5, NaCl concentration increase from 10^{-4} to 10^{-1} mol/L). Symbols – experimental data, lines – fitting with exponential decay. The values of $t_{95\%}$ are shown in the plot. Shaded area indicates the standard deviation (n = 5).

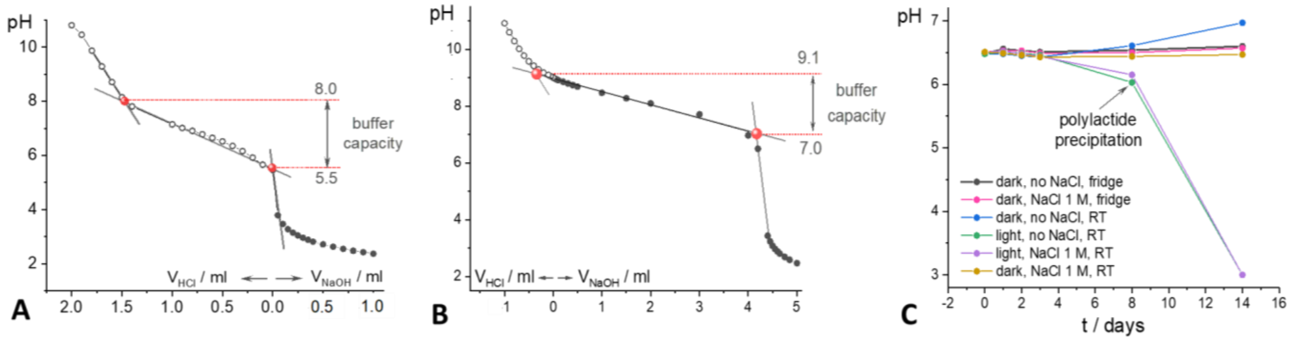

Fig. S8. A, B – potentiometric titration of acidic and alkaline model sweat, open symbols – addition of acid, solid symbols – addition of alkali; C – the stability of pH of the neutral sweat standard over time dependent on various factors (indicated in the plot).

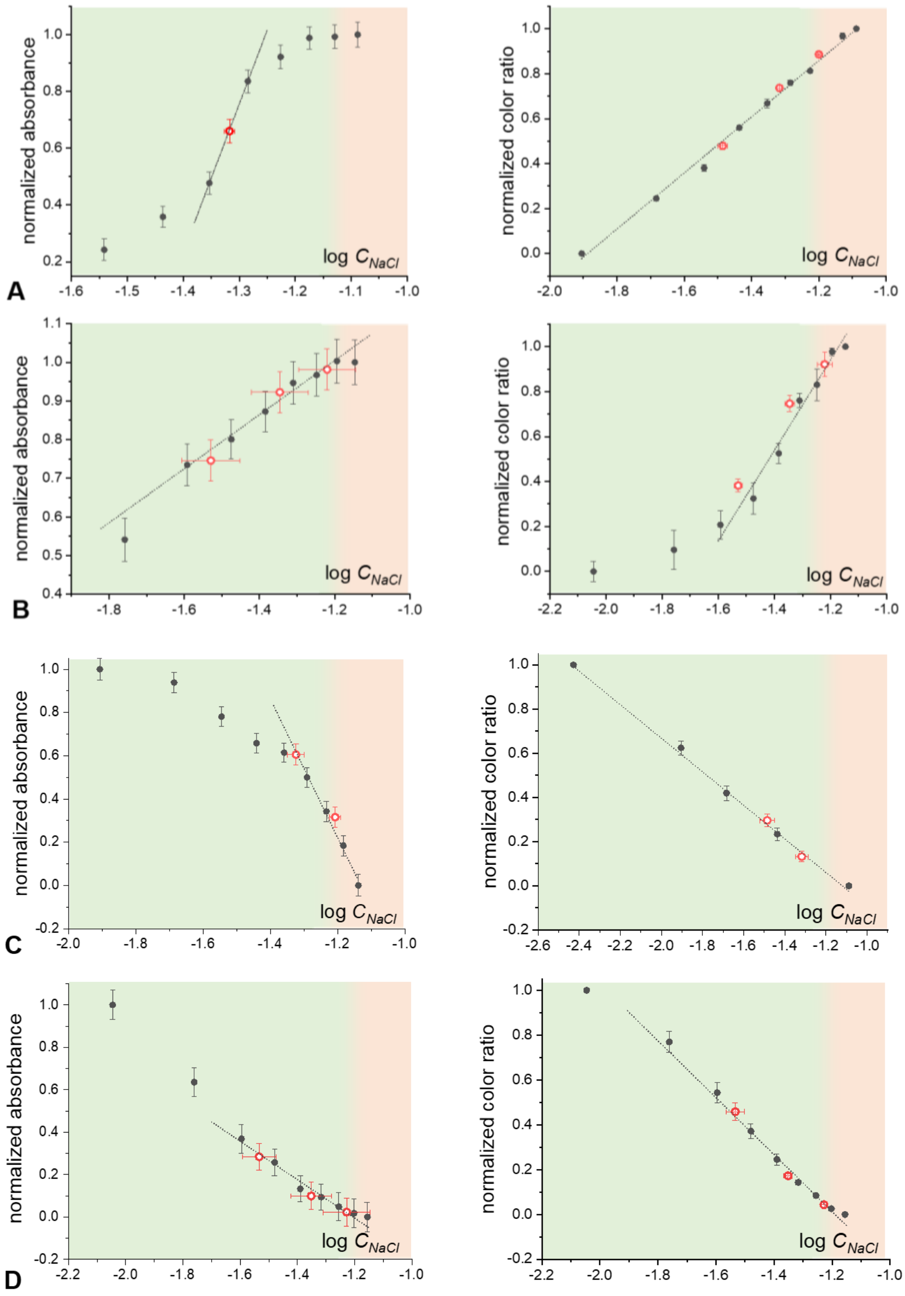

Fig. S9. Calibration curves (solid symbols) of the sensors of: A – composition **1** (pH = 5.5), B – composition **5** (pH = 8.0), C – composition **10** (pH = 5.5), D – composition **12** (pH = 8.0) in artificial sweat solutions. Left panels – spectrophotometric response of thin optode films; right panels – colorimetric response of drop-cast arrays acquired with microscope camera. Open symbols – independently prepared artificial sweat samples used for recovery-based validation. Green range – normal Na^+ and Cl^- concentrations in sweat, orange range – elevated concentrations. The error bars represent indirect measurement error estimated from photometric repeatability (left panels) or standard deviation of the experimental data (right panels, n = 6).

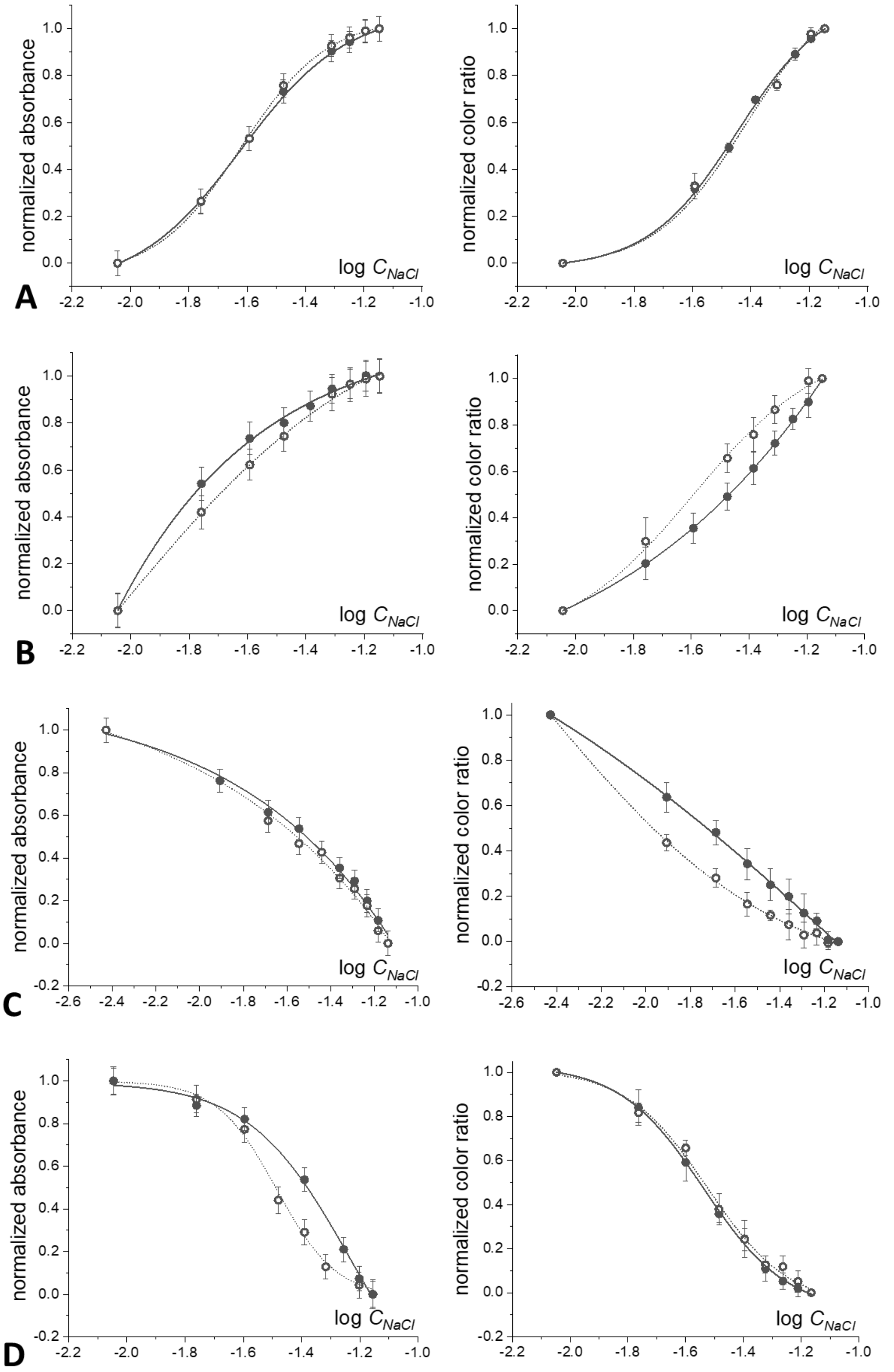

Fig. S10. Hysteresis of the optical response for the sensors of: A – composition **2** (pH = 6.5), B – composition **5** (pH = 8.0), C – composition **10** (pH = 5.5), D – composition **11** (pH = 6.5) in artificial sweat solutions. Left panels – spectrophotometric response of thin optode films (error bars – indirect measurement error estimated from photometric repeatability); right panels – colorimetric response of drop-cast arrays acquired with microscope camera (error bars – standard deviation, n = 6). Solid symbols denote data acquired during stepwise increase of NaCl concentration; open symbols represent the descending concentration branch. Lines – Boltzmann function fitting (Eq. 3).

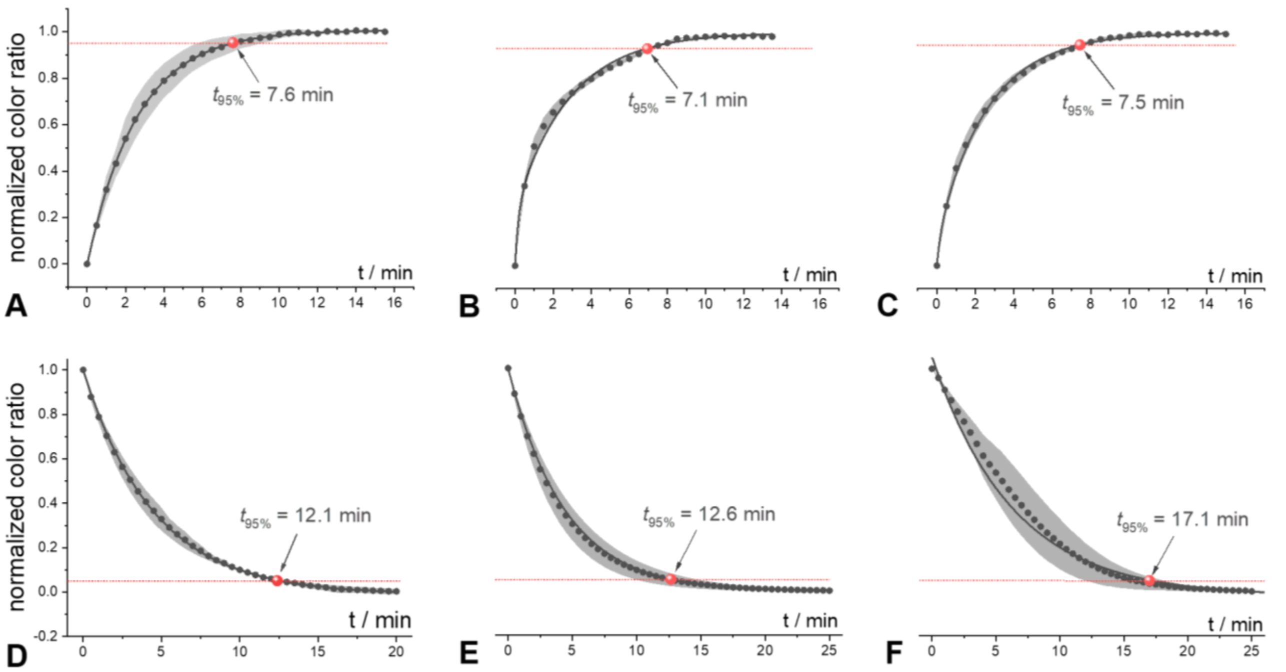

Fig. S11. The dynamic response curves for the drop-cast sensors measured in artificial sweat solutions (A – composition **1**, pH 5.5, B – composition **2**, pH 6.5, C – composition **5**, pH 8.0, D – composition **10**, pH 5.5, E – composition **11**, pH 6.5, F – composition **12**, pH 8.0). NaCl concentration increase from 10^{-4} to 10^{-1} mol/L. Symbols – experimental data, lines – fitting with exponential decay. The values of $t_{95\%}$ are shown in the plots. Shaded areas indicate the standard deviation (n = 6).

Table S5. The results of recovery studies with sodium- and chloride-selective optodes of different compositions, obtained by spectrophotometry and macrophotography with DCA.

**artificial sweat**	**registration method**	**input**		**found**		**recovery, %**	
		**log a_{Na}**	**log a_{Cl}**	**log a_{Na}**	**log a_{Cl}**	**Na**	**Cl**
		comp. **1**	comp. **10**	comp. **1**	comp. **10**		
acidic	macrophotography	-1.49	-1.49	-1.48±0.01	-1.52±0.04	99.8±0.2	97.9±2.6
		-1.32	-1.32	-1.31±0.01	-1.31±0.03	99.2±0.7	99.0±1.9
		-1.20	-1.20	-1.19±0.01	–	99.6±1.1	–
acidic	spectrophotometry	-1.49	-1.49	–	–	–	–
		-1.32	-1.32	-1.32±0.01	-1.40±0.03	99.5±0.9	94.2±2.2
		-1.21	-1.21	–	-1.23±0.02	–	98.3±1.0
		comp. **2**	comp. **11**	comp. **2**	comp. **11**		
neutral	macrophotography	-1.53	-1.53	-1.52±0.02	-1.63±0.02	99.6±1.5	93.9±1.6
		-1.35	-1.35	-1.31±0.03	-1.41±0.02	97.4±2.3	95.8±1.7
		-1.22	-1.23	-1.19±0.02	-1.26±0.02	97.7±1.7	97.0±1.1
neutral	spectrophotometry	-1.53	-1.53	-1.48±0.05	-1.53±0.03	96.3±2.8	99.7±1.7
		-1.35	-1.35	-1.39±0.05	-1.33±0.03	96.5±3.0	97.9±1.6

		-1.22	-1.23	-1.19±0.05	-1.22±0.03	97.5±3.8	99.0±2.2
		comp. **5**	comp. **12**	comp. **5**	comp. **12**		
		-1.53	-1.53	-1.57±0.01	-1.54±0.03	97.1±0.4	99.6±2.2
alkaline	macrophotography	-1.35	-1.35	-1.37±0.02	-1.34±0.02	98.0±0.9	98.9±1.1
		-1.22	-1.23	-1.23±0.03	-1.22±0.02	98.9±2.0	99.0±1.4
		-1.53	-1.53	-1.57±0.08	-1.52±0.06	97.5±5.2	99.1±3.6
alkaline	spectrophotometry	-1.35	-1.35	-1.33±0.08	-1.33±0.07	98.6±6.0	98.6±5.3
		-1.22	-1.23	-1.21±0.08	-1.21±0.07	99.3±6.6	98.5±5.9

References

[1] C. Callewaert, B. Buysschaert, E. Vossen, V. Fievez, T. Van de Wiele, and N. Boon, "Artificial sweat composition to grow and sustain a mixed human axillary microbiome", *Journal of microbiological methods,* vol. 103, pp. 6-8, Aug 2014, doi: 10.1016/j.mimet.2014.05.005.

[2] GOST R ISO 11641-2015, "Leather – Tests for color fastness – Method for determination of color fastness to perspiration", Moscow: Standardinform, 2015.

[3] D. I. Dekina, A. V. Kalinichev, N. V. Pokhvishcheva, M. A. Peshkova, and K. N. Mikhelson, "Effects of quantitative composition of the sensing phase in the response of ionophore-based optical sensors", *Sensors and Actuators B: Chemical,* vol. 277, pp. 535-543, Dec 2018, doi: 10.1016/j.snb.2018.09.018.
